# Early‐life regional and temporal variation in filaggrin‐derived natural moisturizing factor, filaggrin‐processing enzyme activity, corneocyte phenotypes and plasmin activity: implications for atopic dermatitis†

**DOI:** 10.1111/bjd.16691

**Published:** 2018-06-29

**Authors:** M.A. McAleer, I. Jakasa, N. Raj, C.P.F. O'Donnell, M.E. Lane, A.V. Rawlings, R. Voegeli, W.H.I. McLean, S. Kezic, A.D. Irvine

**Affiliations:** ^1^ National Children's Research Centre Our Lady's Children's Hospital Dublin Ireland; ^2^ Paediatric Dermatology Our Lady's Children's Hospital Dublin Ireland; ^3^ Laboratory for Analytical Chemistry Department of Chemistry and Biochemistry Faculty of Food Technology and Biotechnology University of Zagreb Zagreb Croatia; ^4^ School of Pharmacy University College London London U.K.; ^5^ Department of Neonatology National Maternity Hospital Dublin Ireland; ^6^ DSM Nutritional Products Ltd Wurmisweg 571 Kaiseraugst Switzerland; ^7^ Dermatology and Genetic Medicine Division of Biological Chemistry and Drug Discovery School of Life Sciences University of Dundee Dundee U.K.; ^8^ Coronel Institute of Occupational Health Amsterdam Public Health Research Institute Academic Medical Center Amsterdam the Netherlands; ^9^ Department of Clinical Medicine Trinity College Dublin Dublin Ireland

## Abstract

**Background:**

Filaggrin is central to the pathogenesis of atopic dermatitis (AD). The cheeks are a common initiation site of infantile AD. Regional and temporal expression of levels of filaggrin degradation products [natural moisturizing factors (NMFs)], activities of filaggrin‐processing enzymes [bleomycin hydrolase (BH) and calpain‐1 (C‐1)] and plasmin, and corneocyte envelope (CE) maturity in early life are largely unknown.

**Objectives:**

We conducted a cross‐sectional, observational study investigating regional and age‐dependent variations in NMF levels, activity of proteases and CE maturity in stratum corneum (SC) from infants to determine whether these factors could explain the observed predilection sites for AD in early life.

**Methods:**

We measured NMF using a tape‐stripping method at seven sites in the SC of 129 children (aged < 12 months to 72 months) and in three sites in 56 neonates and infants (< 48 h to 3 months). In 37 of these neonates and infants, corneocyte size, maturity, BH, C‐1 and plasmin activities were determined.

**Results:**

NMF levels are low at birth and increase with age. Cheek SC, compared with elbow flexure and nasal tip, has the lowest NMF in the first year of life and is the slowest to reach stable levels. Cheek corneocytes remain immature. Plasmin, BH and C‐1 activities are all elevated by 1 month of age in exposed cheek skin, but not in elbow skin.

**Conclusions:**

Regional and temporal differences in NMF levels, CE maturity and protease activities may explain the predilection for AD to affect the cheeks initially and are supportive of this site as key for allergen priming in early childhood. These observations will help design early intervention and treatment strategies for AD.

## Abstract 2

**What's already known about this topic?**

- Atopic dermatitis (AD) frequently starts in early infancy, and the first eczematous lesions emerge on the cheeks.
- Filaggrin is a major structural protein in the stratum corneum (SC).
- Filaggrin deficiency is associated with the development of AD and, in the context of AD, food allergies and asthma.
- Filaggrin is metabolized into natural moisturizing factors (NMFs), which can be measured in the SC.

**What does this study add?**

- Regional differences in NMF levels, corneocyte envelope immaturity and protease activities may help explain why infantile AD most often initially affects the cheeks.
- Filaggrin processing, corneocyte maturity, and protease activities show regional and temporal differences in infant skin.
- These findings may explain disease patterns in early‐life AD.

**What is the translational message?**

- Cheek skin may be highly relevant for allergen priming.
- Emollient therapy at the vulnerable cheek site might help to prevent AD and/or food sensitization.

**Linked Editorial:** https://doi.org/10.1111/bjd.16806.

https://doi.org/10.1111/bjd.16959 available online

https://goo.gl/Uqv3dl

Atopic dermatitis (AD) is the most common chronic inflammatory disease of childhood in the developed world.^1^ Clinical manifestations of AD vary with age. In infancy, the first lesions most commonly emerge on the cheeks and the scalp.^2^ A Danish study of 411 infants reported that AD lesions were found to begin on the scalp, forehead, ear, neck and cheek in infants, with the cheek being the most commonly involved region.^3^ While infantile AD typically involves the facial skin, the nasal tip is almost always lesion free (the ‘Yamamoto’ sign).^4^ These well‐recognized predilection sites for AD suggest distinct regional differences in skin structure and function.

Structural differences between infant and adult skin have been reported.^5^, ^6^ Infant corneocytes are smaller than those of adults, correlating with a higher epidermal cell turnover rate compared with adults.^7^ In contrast to adults, newborn babies do not show variation in corneocyte size between nonacral skin regions. Their corneocytes are uniformly small and are similar in size to those found in the adult forehead.^8^ This suggests consistently high epidermal turnover across the entire skin surface in neonates. Adaptive changes, both in stratum corneum (SC) water‐handling properties and skin surface pH, occur in early life. In full‐term neonates, transepidermal water loss (TEWL) data suggest a competent epidermal barrier function shortly after birth.^5^, ^9^

A defective skin barrier is a key feature of AD.^10^ Filaggrin is a major structural protein in the SC and its constituent amino acids play a critical role in SC acidification and hydration. Filaggrin deficiency is associated with impaired barrier function and the development of AD.^11^ Filaggrin deficiency in children seems to define a specific endotype of AD, characterized by a predilection to exposed areas of the body, in particular the cheeks and hands.^12^ Profilaggrin, the phosphorylated precursor to filaggrin, is expressed in the upper stratum granulosum, where processing is initiated. A complex cascade of dephosphorylation and proteolysis liberates functionally active filaggrin monomers that, within the SC, are further cleaved into short peptides and are finally degraded fully to form a cytoplasmic pool of hygroscopic amino acids and their derivatives, known as natural moisturizing factor (NMF). Following deimination of arginine residues, the cysteine/aspartic proteases caspase‐14, bleomycin hydrolase (BH) and calpain‐1 (C‐1), located mainly in the upper SC, have been shown to be important in enzymatic degradation of filaggrin.^13^, ^14^ C‐1 is also involved in the processing of profilaggrin to filaggrin, and also in corneocyte maturation.^15^, ^16^

NMF modulates the pH of the SC surface, promotes the retention of water within the corneocytes and may exert antimicrobial activity.^1^ Important amino acid derivatives include *trans*‐urocanic acid (t‐UCA) and pyrrolidone carboxylic acid (PCA).^1^ The enzyme histidase converts histidine (His) to t‐UCA.^17^ Glutamic acid is converted to PCA, by nonenzymatic degradation (cyclization) in addition to enzymatic processes and overall, constitutes 12% of NMF.^18^, ^19^, ^20^ In addition, non‐filaggrin‐derived compounds such as lactate, urea, sugars and ions contribute to NMF. The amino acids and their derivatives that constitute NMF can be measured and closely correlate with filaggrin content.^21^ The epidermis expresses several proteases that are involved in a range of key epidermal responses, including proliferation, differentiation, lipid barrier homeostasis and tissue remodelling.^22^ Plasmin plays an important, but still not completely defined role in the corneocyte maturation process and is a marker for SC stress and inflammation, even in areas lacking clinically observable inflammation.^23^

The reasons why AD has a predilection for certain body sites and why the affected regions vary with age in childhood remain unknown. Age‐related and regional differences in skin structure and function are likely important. Here, we investigated the regional and temporal expression of filaggrin‐derived NMF together with filaggrin‐processing enzyme activities (BH and C‐1), corneocyte phenotypes and plasmin activities in a large cohort of healthy neonates, infants and children in anatomical locations relevant for AD.

## Patients and methods

### Study population

In phase I of the study, healthy infants and children were recruited from March to September 2013. These infants and children were scheduled to undergo elective surgical procedures in Our Lady's Children's Hospital, Dublin, Ireland. Children were recruited if they did not have a history suggestive of AD or any other inflammatory skin disease. An experienced paediatric dermatologist (M.A.McA.) examined all infants. Other exclusion criteria from the study included infants and children who had pyrexial illness in the preceding 2 weeks, those who had received immunosuppressive systemic therapy, such as oral corticosteroids, in the preceding 3 months, and patients whose ancestry was not exclusively Irish (four of four grandparents). Non‐Irish children were excluded to allow for accurate *FLG* mutation stratification. The following seven body sites were tape stripped in phase I: cheek, nasal tip, nape of neck, volar forearm at the elbow flexure, dorsal proximal mid‐upper limb, dorsal hand and the buttock.

Phase II of the study was carried out in The National Maternity Hospital, Holles Street, Dublin, Ireland (from July to September 2014). Infants were recruited, both in the postnatal wards in the 48 h following delivery and in the early weeks of life in outpatient/ambulatory clinics, when attending for elective baby checks. Identical inclusion and exclusion criteria for phase I were applied for phase II. The following three body sites were tape stripped in this cohort: cheek (C), nasal tip (T) and volar forearm at the elbow flexure (E). The number of body sites sampled had to be reduced in view of the very young age of these individuals. The three body sites in this cohort were selected because preliminary data from phase I of the study suggested that these body sites were most likely to provide the greatest amount of information. As this was an exploratory study, a definitive sample size that would allow statistically significant findings was difficult to predict. After phase I yielded significant results, we calculated that the smaller sample size would be informative in phase II. The power analysis was based on the difference in NMF values obtained between skin sites C, T and E. The highest sample size of *n* = 38 (power 80%; *P* = 0·05, paired *t*‐test) was obtained for the difference between C and E (mean 0·21 and SD 0·47). We included more children (*n* = 59) as we expected that tape stripping might fail in neonates and infants.

The study was conducted in accordance with the Declaration of Helsinki principles and was approved by the Research Ethics Committee of Our Lady's Children's Hospital, Dublin, Ireland. Full written informed consent was obtained from the parents of each individual.

### Sampling of the stratum corneum by tape stripping

All sampled sites were emollient free for 24 h before tape stripping. The SC was sampled using the method previously described.^21^ Circular adhesive tape strips (3·8 cm^2^, D‐Squame, CuDerm, Dallas, TX, U.S.A.) were attached to the skin and, in all regions apart from the nasal tip, pressed for 10 s with a constant pressure (225 g cm^−2^) using a D‐Squame pressure instrument D500 (CuDerm). On the nasal tip, the circular adhesive tape strip was cut in half, placed on the skin, and pressure was applied manually for 10 s.^24^ Eight consecutive tape strips were sampled, all from the same site. Tape strips were stored at −80 °C until analysis.

### Filaggrin genotyping

All patients were screened for the nine most common *FLG* mutations found in the Irish population (R501X, Y2092X, 2282del4, R2447X, S3247X, R3419X, 3702X, S1040X and G1139X) from DNA extracted from a blood sample or buccal swab. The methods used have been previously described.^25^ All individuals that were heterozygous or homozygous for loss‐of‐function mutations in the *FLG* gene were excluded from the analysis.

### Determination of filaggrin breakdown products in the stratum corneum

NMF component analysis (His, PCA, *trans*‐UCA and *cis*‐UCA) was performed and total SC protein determined on the fourth consecutive tape stripping according to the method described in detail elsewhere.^21^ The four components were combined to give a ‘total NMF’ value. NMF components in the SC from each tape stripping were extracted with 500 μL 25% (*w/w*) ammonia solution. After evaporation of the ammonia extract, the residue was reconstituted in 250 μL pure water and analysed by high‐performance liquid chromatography ultraviolet analysis. Owing to incomplete extraction recovery of SC proteins by ammonia, the second extraction of proteins from the tape stripping was performed with 0·1 mol L^−1^ KOH solution over 24 h. The proteins were determined in both extracts by using Pierce Micro BCA protein assay kit (Thermo Fisher Scientific, Rockford, IL, U.S.A.). The levels of NMF components in the SC were normalized for proteins and expressed as mmol NMF per g of protein.

### Natural moisturizing factor depth profiling

To ensure that NMF measurement in different age groups and sites was not affected by varying SC thickness, NMF profiling was performed on consecutive tape strips in 13 individuals. NMF was assessed in the second, sixth and eighth tape stripping in participants from < 48 h to 3 months of age at the cheek and elbow sites.

### Stratum corneum maturation assays

The first tape stripping from 37 individuals at two skin sites, i.e. the cheek and elbow, were investigated for corneocyte envelope (CE) maturity. This was performed using differential Nile red and immunostaining for the late epidermal differentiation marker involucrin, using a modification of the previously described method.^26^

### Measurement of stratum corneum protease activities: calpain‐1 and bleomycin hydrolase and plasmin

The same 37 individuals were assessed for plasmin, C‐1 and BH activities. Protease activities were determined on the fifth tape stripping using previously reported methods.^23^, ^26^, ^27^, ^28^, ^29^

### Statistical analysis

All calculations were performed using Prism 6 software (GraphPad, San Diego, CA, U.S.A.). The distribution of data was tested using the Shapiro–Wilk normality test. The applied statistical test is indicated in the figure legends.

## Results

### Demographic data of participants

In total, 188 infants and children were recruited; 112 were male and 76 were female (Table 1). The average age, age range and age groups are outlined in Tables 1 and 2. The demographic characteristics of the 37 participants for whom corneocyte maturity assays were performed and measurements of C‐1, BH and plasmin were taken are outlined in Table S1 (see Supporting Information). *FLG* mutation status was determined for all participants in phase I. In phase II we were unable to establish the *FLG* status definitively in 12 participants because of poor DNA quality obtained from this subset of buccal swabs (Table S2; see Supporting Information). These were excluded from analysis.

**Table 1:** Age groups and sex of all recruited participants

	*N*	Male	Female	Mean age	Age range
Phase I	129	78	51	27·9 months	0·25–70 months
Phase II	59	34	25	20·5 days	1–84 days
Total	188	112	76		

**Table 2:** Sex distribution, mean age and age range of all participants in phase I and phase II of study

Age groups	< 48 h	48 h to 4 weeks	1–3 months	4–11 months	12–36 months	36–72 months
Male, *n*	13	12	17	14	28	27
Female, *n*	13	8	9	14	16	16
Total	26	20	26	28	44	43

### Total natural moisturizing factor is low after birth, increases rapidly in postnatal life and is regionally determined, with the cheek skin being slow to mature

**Figure 1:**
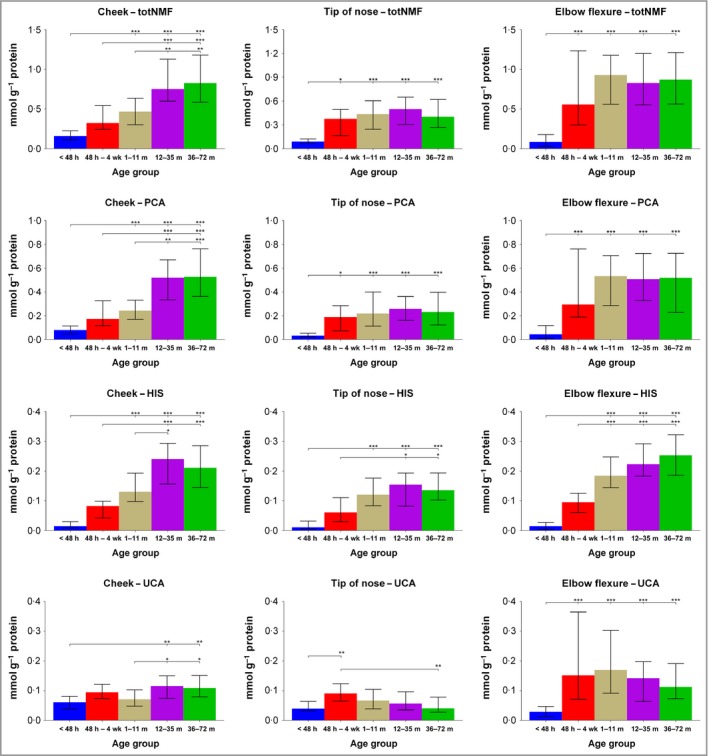
Levels of total natural moisturizing factor (NMF), histidine (His), pyrrolidone carboxylic acid (PCA) and sum of *trans*‐urocanic acid (UCA) and *cis*‐UCA in the stratum corneum of children (median with interquartile range) in different age groups [< 48 h (*n* = 18), 48 h – 4 weeks (*n* = 18), 1–11 months (*n* = 41), 12–35 months (*n* = 25) and 36–72 months (*n* = 30)] on three body regions. Differences between age groups were determined by Kruskal–Wallis test followed by Dunn's multiple comparisons test. ***P < 0.001, **P < 0.01 and *P < 0.05.

In the first 48 h after birth, the total NMF levels were low in all sampled regions. There was an increase in the total NMF levels in the early weeks of life in all regions. The nasal tip and the elbow flexure had a rapid increase in total NMF levels in the first 4 weeks of life, after which NMF stabilizes, with no further increase (Fig. 1). In contrast, in cheek skin there was still a significant increase (*P* < 0·001, Mann–Whitney two‐sided paired test) in NMF between the time periods of 1–11 months and 12–35 months. Furthermore, the rate of increase in levels of NMF in cheek SC was much slower (Fig. S1; see Supporting Information), and there was a significant (*r* 2 = 0·28, *P* < 0·001) linear response of NMF vs. age from 1 month up to 72 months (Fig. S1; see Supporting Information). Of note, there was no variation in NMF levels with SC depth after 48 h of age (Fig. S2; see Supporting Information). NMF levels on the remaining body sites sampled in phase I (nape of neck, dorsal hand, dorsal upper limb and buttock) were stable from 1 month to 72 months of age (Fig. S3; see Supporting Information). In the first year of life, the NMF values for the cheek SC were significantly lower compared with those of the elbow (median of difference 0·15; *P* < 0·001, Wilcoxon paired *t*‐test), although this difference was not significant at ages > 12 months (Fig. S4; see Supporting Information).

### The histidine/urocanic acid ratio and the *trans*‐urocanic acid/*cis*‐urocanic acid ratio change rapidly postnatally and transition to a steady state more quickly in exposed skin sites compared with nonexposed sites

**Figure 2:**
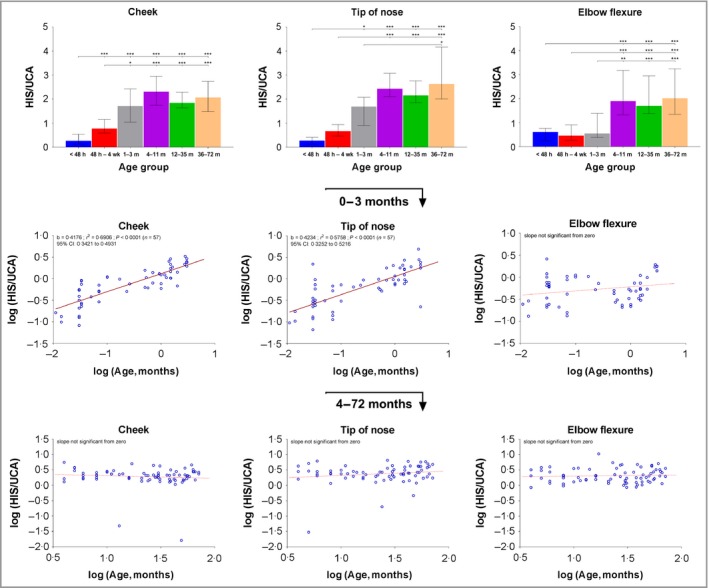
Ratio of histidine (His) to urocanic acid (UCA) (median with interquartile range) (a–c) and linear regression between ratio of His to UCA and age of children [(d–f) up to 3 months of age; (g–i) from 3 to 72 months of age] on three body regions. For regression analysis the values were log‐transformed. Age groups: < 48 h (*n* = 18), 48 h – 4 weeks (*n* = 18), 1–3 months (*n* = 21), 4–11 months (*n* = 20), 12–35 months (*n* = 25), 36–72 months (*n* = 30). ***P < 0.001, **P < 0.01 and *P < 0.05 as determined by Kruskal–Wallis test followed by Dunn's multiple comparisons test. b, slope of the regression line; CI, confidence interval.

**Figure 3:**
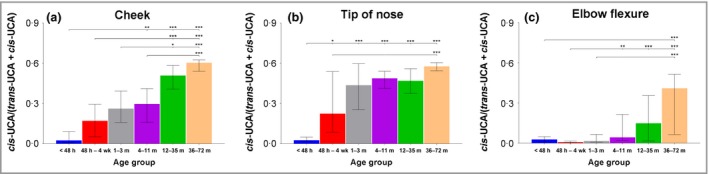
Ratio of *cis*‐urocanic acid (UCA) to total UCA (*cis*‐UCA *+ trans*‐UCA) (median with interquartile range). Age groups: < 48 h (*n* = 18), 48 h – 4 weeks (*n* = 18), 1–3 months (*n* = 21), 4–11 months (*n* = 20), 12–23 months (*n* = 1), 24–35 months (*n* = 14), 36–47 months (*n* = 12) and 48–72 months (*n* = 18). Differences between age groups were determined by Kruskal–Wallis test followed by Dunn's multiple comparisons test. ***P < 0.001, **P < 0.01 and *P < 0.05.

The His/UCA ratio rapidly increases in the first 3 months of life in exposed sites, and reaches a steady state from 4 months of age onwards (approximately twofold to threefold). In the nonexposed elbow flexure skin the His/UCA ratio remains low for the first 3 months of life (Fig. 2). The *cis*‐UCA/total UCA ratio increases rapidly in postnatal life in exposed sites. The ratio of *cis*‐UCA to the total amount of UCA (*cis* + *trans* isomer) in the cheek and nasal tip skin reaches the levels 30–50% around the first year of life while the ratio between *cis*‐UCA/total UCA in the elbow flexure SC remains low (Fig. 3).

### Bleomycin hydrolase and calpain‐1 activities increase after 1 month of life and are higher in the cheek than the elbow

**Figure 4:**
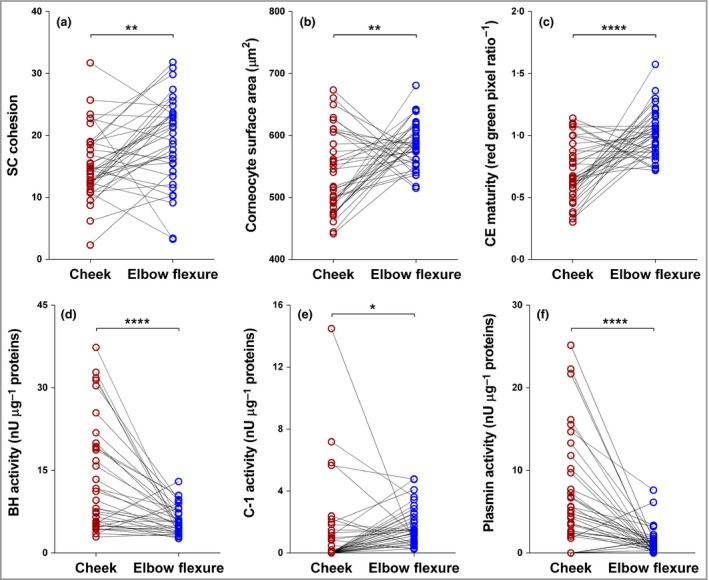
(a–c) Corneocyte envelope (CE) maturity, stratum corneum (SC) cohesion, cornecyte surface area and activity of (d–f) bleomycin hydrolase (BH), calpain‐1 (C‐1) and plasmin in the SC of children up to 11 months of age (*n* = 37). Difference between cheek region and elbow flexure region were determined by two‐tailed paired *t*‐test (SC cohesion and CE maturity) or by two‐tailed Wilcoxon matched‐pairs signed‐rank test (corneocyte surface area, BH, C‐1 and plasmin). ****P < 0.0001, **P < 0.01.

The late‐stage filaggrin‐processing enzymes, BH and C‐1 activities are similar in the cheek and elbow skin after birth. After 1 month of age the levels increase significantly in the exposed cheek skin, but not at the elbow (Figs 4 and S5; see Supporting Information).

### Plasmin activity increases at 1 month of age in the cheek and correlates with *trans*‐urocanic acid/*cis*‐urocanic acid ratio in cheek skin

**Figure 5:**
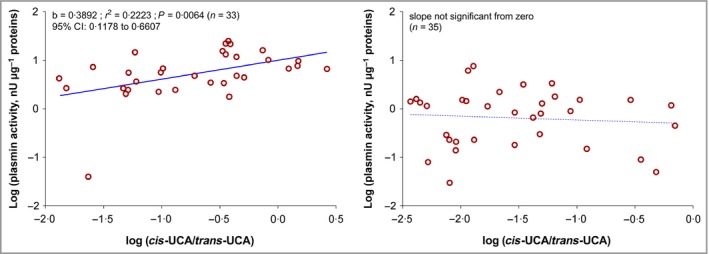
Linear regression plasmin activity vs. ratio of *cis*‐urocanic acid (UCA) to total UCA (*cis*‐UCA *+ trans*‐UCA) on two body sites. The values were log‐transformed. b, slope of the regression line; CI, confidence interval.

Plasmin activities are higher in the cheek compared with the elbow at birth (Fig. 4). At 1 month, the plasmin activity generally increases in the exposed cheek skin. Plasmin activities correlate with *trans*‐UCA/*cis*‐UCA ratios at the cheek (Fig. 5).

### Cheek corneocytes have a more immature phenotype in contrast with elbow corneocytes

**Figure 6:**
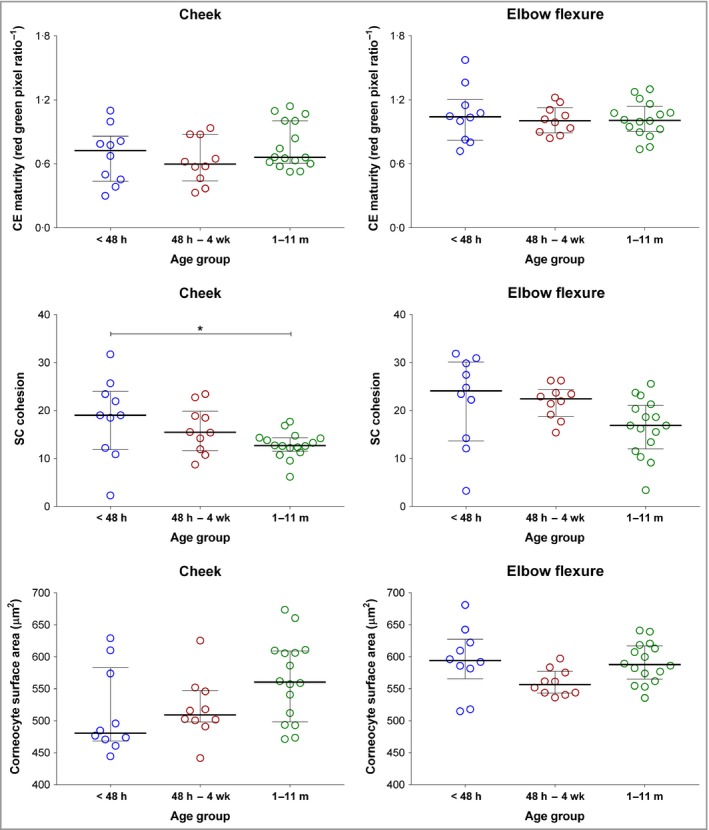
Corneocyte envelope (CE) maturity, stratum corneum (SC) cohesion and cornecyte surface area in the SC of children (median with interquartile range) in three age groups across two body regions. Age groups: < 48 h (*n* = 10); 48 h – 4 weeks (*n* = 10) and 1–11 months (*n* = 16). Differences between age groups were determined by one‐way anova followed by Tukey's multiple comparison test (for CE maturity and cornecyte surface area on cheek region Kruskal–Wallis test followed by Dunn's multiple comparisons test was used). *P < 0.05.

The cheek corneocytes have an immature phenotype from birth, which does not change with age. This contrasts with the elbow corneocytes, which have a mature phenotype from birth (Fig. 4). In keeping with an immature phenotype, the cheek corneocytes had higher cohesion – specifically, less SC protein was removed, which meant that the corneocytes were more tightly cohesive (Fig. 6).

## Discussion

Our results show that NMF is low at birth at all investigated skin sites and increases significantly over the first month of life, suggesting that the SC rapidly adapts to the dramatic environmental shift from *in utero* to *ex utero*. Our findings are in keeping with a previous study that measured free amino acids in the chest or back skin of 30 neonates and infants.^30^ Similarly, Chittock *et al*. reported low NMF in the skin of 115 neonates at birth, with a significant increase in NMF by 4 weeks of age.^31^ Low NMF in infant skin at birth is in keeping with reports in variations between neonatal and adult rat skin. Scott and Harding reported that during late fetal development filaggrin accumulates throughout the entire thickness of rat SC and immediately after birth filaggrin proteolysis occurs in the outer SC.^32^

**Figure 7:**
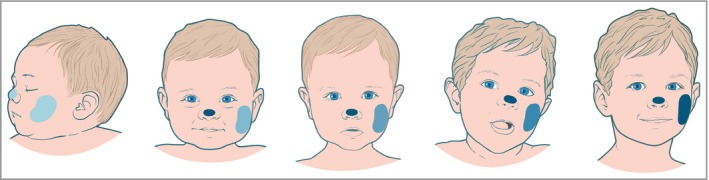
Cheek skin is slow to mature with respect to natural moisturzing factor (NMF) levels and corneocyte maturity. While other exposed sites, such as the nasal tip, rapidly reach steady‐state maturity in the early months of life, the cheek skin starts life with the lowest NMF and only reaches steady‐state NMF levels at approximately 7 years of age. NMF levels are graphically represented by depth of blue colour.

We found that the cheek skin was a unique site with respect to NMF levels and corneocyte maturity in infancy. NMF levels in cheek SC were much slower to increase compared with other sites, including exposed sites such as the nasal tip (Fig. 7). Furthermore, in the first year of life the cheek NMF levels were significantly lower than the corresponding levels in the elbow SC. The cheek is frequently the site of initial inflammation in infantile AD. Absolute low levels of NMF and a slow increase in NMF levels may play a role in disease initiation at this site, as may high plasmin activity. The elbow flexure was notable for reaching a steady state of NMF early in infancy. This site is not typically involved in infantile eczema, but is a classic site of childhood AD. The reason why this site is vulnerable in childhood AD is unclear, but may reflect the local microbiome rather than NMF levels. It has been shown that age strongly affects the microbiome in infants, with bacterial community structure and diversity shifting over time.^33^ Furthermore, compared with exposed skin sites, elbow flexure skin had a consistently lower ratio of *cis*‐UCA to total UCA. *Cis*‐UCA has previously been suggested as an immunosuppressant.^34^

We also report other important insights into postnatal SC biology. In early postnatal life, there is a rapid increase in the ratio between His and its metabolite UCA (Fig. 2), which levels off after approximately 3 months. This might be caused by the differences related to age and skin site in the expression and activity of histidase, which has an optimum pH of about 8·5 and decreases towards a lower pH.^35^ The pH of the skin surface is highest in the first 2 weeks after birth (pH = 6·0) and gradually decreases with age.^36^

To provide a more comprehensive definition of the generation of NMF in the SC in early postnatal life, along with SC stress, we measured the activities of SC proteases BH and C‐1 and plasmin at the cheek and elbow sites in a selection of participants. The active forms of BH and C‐1 are critical in the final stages of filaggrin degradation.^14^, ^15^, ^16^, ^37^, ^38^, ^39^ We demonstrated increased activity of BH in the exposed cheek skin after 1 month of life. In contrast, levels at the elbow remained unchanged with increasing age. At birth plasmin activities, which indicate SC stress and impaired barrier function, are high in the cheek compared with the elbow. At 1 month of age plasmin activities increase in the exposed cheek skin, but not at the elbow. Furthermore, plasmin activity correlated with the *cis*‐UCA/total UCA ratio in the cheek skin, but not at the elbow. Raj *et al*. hypothesized that these atmospheric conditions result in a feedback mechanism to upregulate filaggrin degradation proteases, thereby producing NMF in an effort to improve barrier function and/or epidermal water retention.^29^ Our data support this hypothesis, in addition to demonstrating that it is relevant in infant skin during early postnatal life.

CE maturity is critical for skin barrier function. We show that the cheek CE is immature from birth and does not improve rapidly with age. Therefore, cheek CE immaturity is present from birth and persists into adulthood.^29^, ^40^, ^41^ In adults, facial TEWL is much higher than that of the forearm and upper arm.^42^ It is notable that the facial skin, unlike most other body sites, is continually exposed to environmental stress. The nasal tip is a site of interest in this study; while this site matures rapidly to steady‐state NMF levels, these levels settle at relatively low levels, suggesting that at this site factors other than NMF or filaggrin expression protect against the development of AD. At this site sebum production is much higher, as is skin hydration, which are factors that may offset low NMF.

Furthermore, low NMF and low maturity of the CE in the infant cheek SC may be important for allergen sensitization at this site, and subsequent food allergy. There is clear evidence that epicutaneous exposure to peanut through an impaired skin barrier increases the risk of peanut sensitization and confirmed peanut allergy.^43^, ^44^, ^45^ Early‐life exposure to peanut antigen in household dust is a risk factor for peanut sensitization and allergy in children^46^ and murine models support the concept of an impaired skin barrier as a route for sensitization.^43^, ^44^, ^45^, ^47^ The demonstration of low NMF levels, a more inflammatory plasmin profile and decreased corneocyte maturity in the exposed cheek skin of infants supports the hypothesis of this site being key to food sensitization and allergy in early life, either independently or in the setting of AD. Saliva and food protein exposure causing irritation at this site are likely cofactors in this pathology.

The early postnatal weeks are particularly dynamic for SC maturation. The infant cheek SC has delayed NMF normalization, higher cohesion and reduced CE maturation, and shows evidence of increased SC stress from birth. We propose that this combination of factors facilitates the initiation of AD inflammation and allergic sensitization. Our results support the concept of improving SC moisturization and skin barrier function in sites of vulnerability in infants in an effort to prevent the onset of AD.^48^, ^49^ The use of moisturizers may compensate for the decrease in NMF levels. Even small increases in filaggrin copy number that drive NMF have been shown to be protective against developing AD.^50^

In summary, regional SC biochemical and cellular characteristics may explain the initial clinical patterns of AD in infancy. Cheek SC early in life is likely permissive for the development of AD, allergen penetration and food allergy.

## Supporting information


**Table S1** Demographics of participants included in stratum corneum protease and corneocyte envelope maturity study.

**Table S2** Filaggrin status of all recruited participants.

**Fig S1.** Levels of total natural moisturizing factor (NMF) in the stratum corneum (SC) of children (median with interquartile range) (a–c) and regression analysis of total NMF vs. age of children [up to 4 weeks of age (d–f); from 1 to 72 months of age; panels (g–i)] on three body regions.

**Fig S2.** Level of total natural moisturizing factor (NMF) at different stratum corneum (SC) depth (mean + SEM) in different age groups [< 48 h (*n* = 7) and 48 h to 4 weeks (*n* = 2) and 1–11 months (*n* = 4)] on two body regions, cheek and elbow flexure (depth: 2 = second SC tape, 6 = sixth SC tape, 8 = eighth SC tape).

**Fig S3.** Levels of total natural moisturizing factor (NMF), histidine, pyrrolidone carboxylic acid (PCA) and sum of *trans*‐ and *cis*‐urocanic acid (UCA) in the stratum corneum (SC) of children (median with interquartile range) across four body regions in the following different age groups: 1–11 months (*n* = 25); 12–35 months (*n* = 25) and 36–72 months (*n* = 27).

**Fig S4.** Natural moisturizing factor (NMF) values between cheek (C) and elbow flexure (E) during the first year of life and for all ages > 1 year.

**Fig S5.** Bleomycin hydrolase (BH), calpain‐1 (C‐1) and plasmin activities in the stratum corneum (SC) of children (median with interquartile range) across two body regions in the following three age groups: < 48 h (*n* = 10); 48 h to 4 weeks (*n* = 10) and 1–11 months (*n* = 16).

**Powerpoint S1.** Journal Club Slide Set.

**Video S1** Author Video.
