# Supplementary figures and images for: Early‐life regional and temporal variation in filaggrin‐derived natural moisturizing factor, filaggrin‐processing enzyme activity, corneocyte phenotypes and plasmin activity: implications for atopic dermatitis
Source: Br J Dermatol. 2018 Jun 29;179(2):431–41. doi: 10.1111/bjd.16691 (PMC6175251; doi:10.1111/bjd.16691)

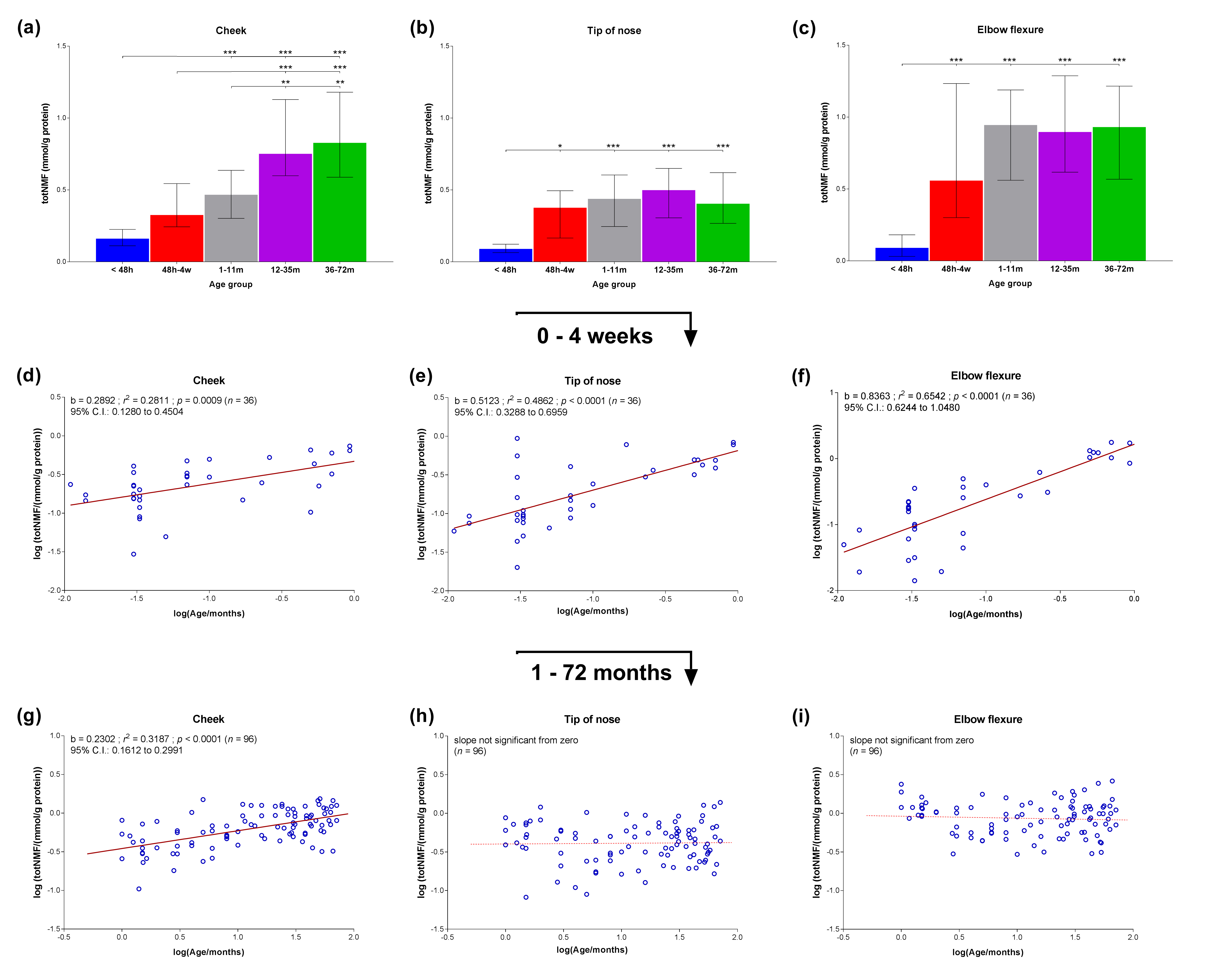

Supplement: Supplementary file 1 — Table S1 Demographics of participants included in stratum corneum protease and corneocyte envelope maturity study. [file BJD-179-431-s001.jpg]

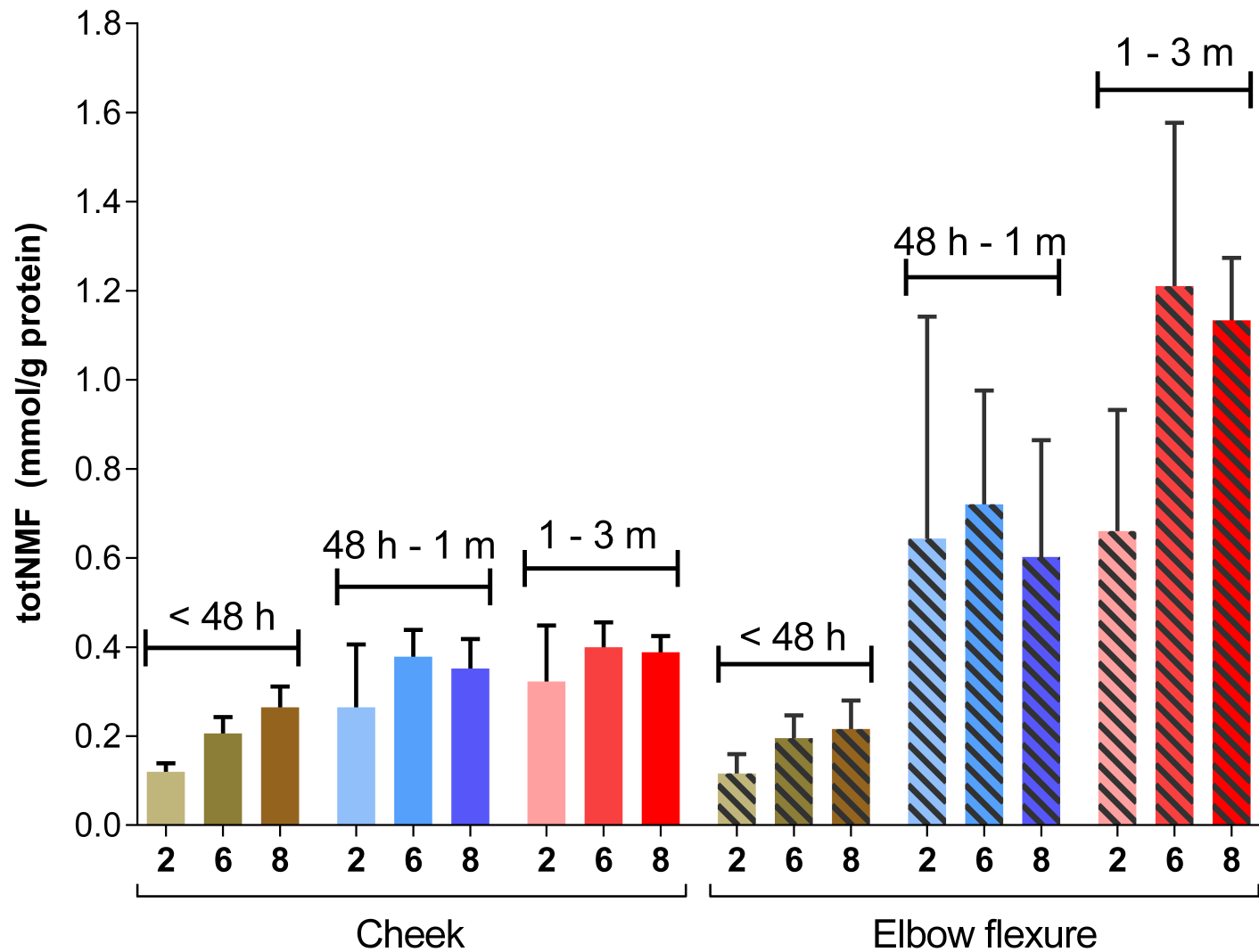

Supplement: Supplementary file 2 — Table S2 Filaggrin status of all recruited participants. [file BJD-179-431-s002.pdf]

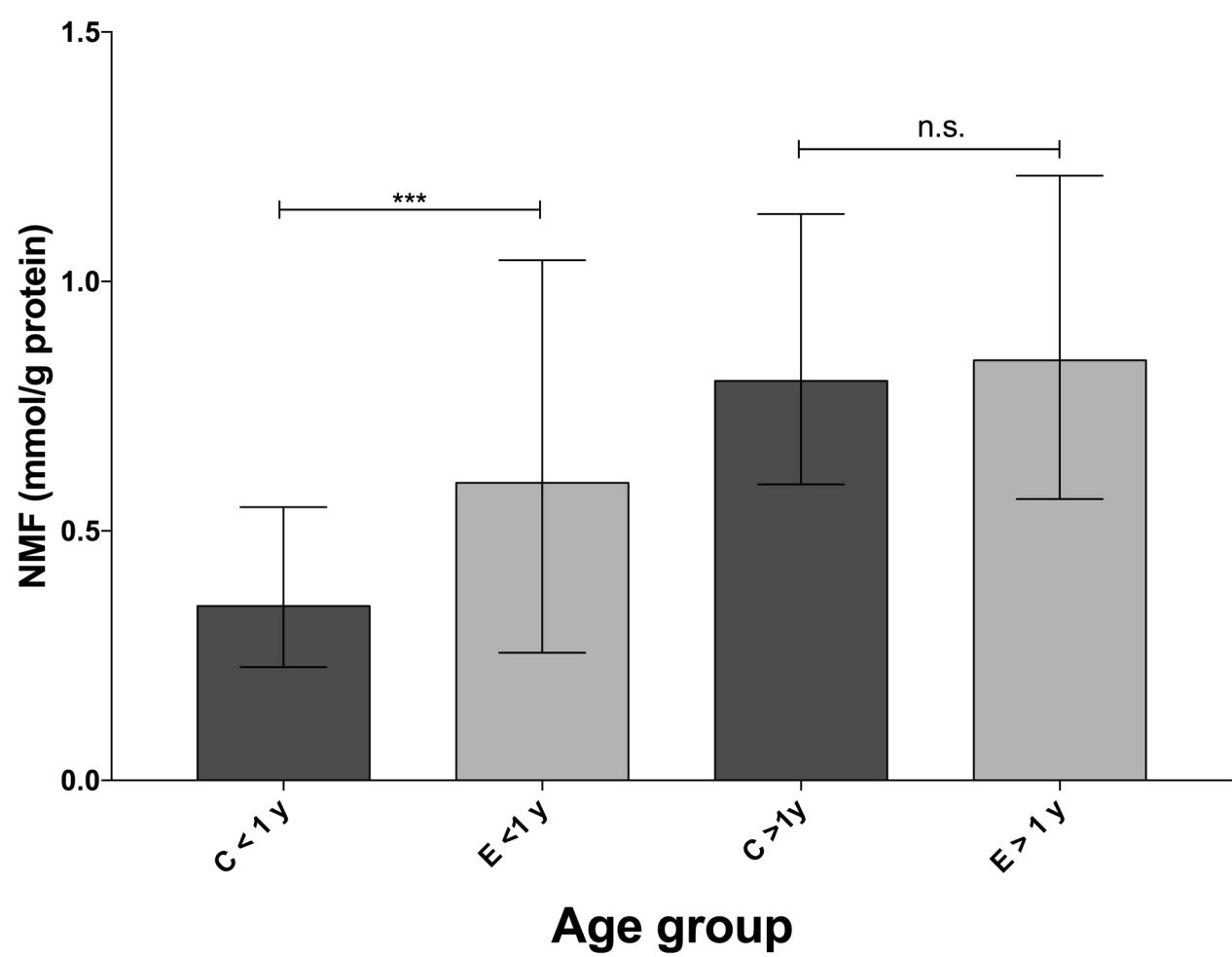

Supplement: Supplementary file 4 — Fig S2. Level of total natural moisturizing factor (NMF) at different stratum corneum (SC) depth (mean + SEM) in different age groups [< 48 h (n = 7) and 48 h to 4 weeks (n = 2) and 1–11 months (n = 4)] on two body regions, cheek and elbow flexure (depth: 2 = second SC tape, 6 = sixth SC tape, 8 = eighth SC tape). [file BJD-179-431-s004.pdf]
