# Supplementary material for: Early‐life regional and temporal variation in filaggrin‐derived natural moisturizing factor, filaggrin‐processing enzyme activity, corneocyte phenotypes and plasmin activity: implications for atopic dermatitis
Source: Br J Dermatol. 2018 Jun 29;179(2):431–41. doi: 10.1111/bjd.16691 (PMC6175251; doi:10.1111/bjd.16691)
Supplement: Supplementary file 3 — Fig S1. Levels of total natural moisturizing factor (NMF) in the stratum corneum (SC) of children (median with interquartile range) (a–c) and regression analysis of total NMF vs. age of children [up to 4 weeks of age (d–f); from 1 to 72 months of age; panels (g–i)] on three body regions. [file BJD-179-431-s003.pdf]

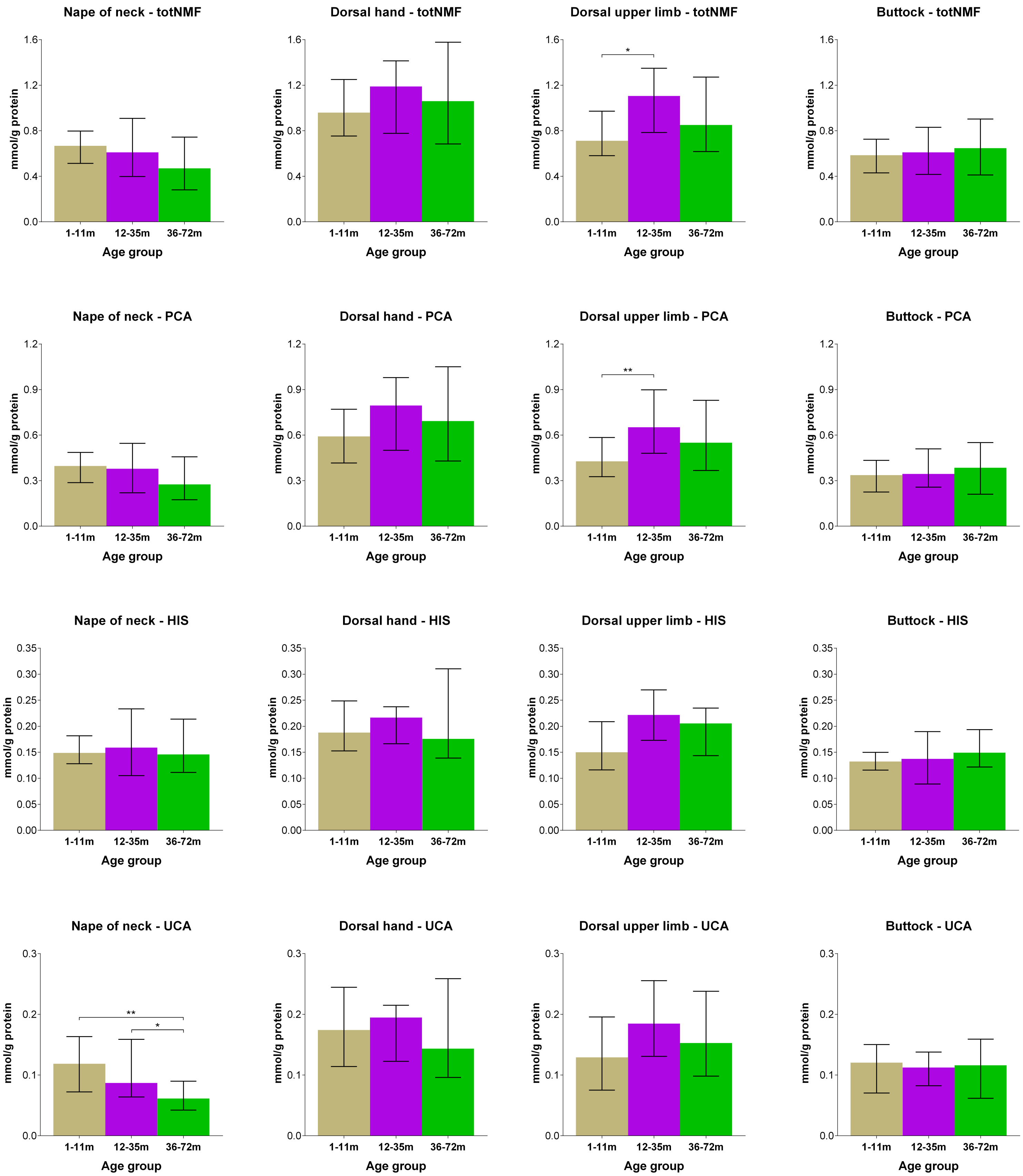

Nape of neck - totNMF
Dorsal hand - totNMF
Dorsal upper limb - totNMF
Buttock - totNMF
Nape of neck - PCA
Dorsal hand - PCA
Dorsal upper limb - PCA
Buttock - PCA
Nape of neck - HIS
Dorsal hand - HIS
Dorsal upper limb - HIS
Buttock - HIS
Nape of neck - UCA
Dorsal hand - UCA
Dorsal upper limb - UCA
Buttock - UCA
mmol/g protein
Age group
1-11m
12-35m
36-72m
0.0
0.4
0.8
1.2
1.6
0.3
0.6
0.9
0.00
0.05
0.10
0.15
0.20
0.25
0.30
0.35
0.1
0.2
*
**
