# Supplementary material for: Early‐life regional and temporal variation in filaggrin‐derived natural moisturizing factor, filaggrin‐processing enzyme activity, corneocyte phenotypes and plasmin activity: implications for atopic dermatitis
Source: Br J Dermatol. 2018 Jun 29;179(2):431–41. doi: 10.1111/bjd.16691 (PMC6175251; doi:10.1111/bjd.16691)
Supplement: Supplementary file 6 — Fig S4. Natural moisturizing factor (NMF) values between cheek (C) and elbow flexure (E) during the first year of life and for all ages > 1 year. [file BJD-179-431-s006.docx]

| Age groups | <48 hrs | 48hr-4w | 1 -3 m | 4-11m | All Age Groups |
| --- | --- | --- | --- | --- | --- |
| Number | 10 | 10 | 9 | 8 | 37 |
| Mean Age (months) | 0.03 | 0.38 | 1.78 | 7.4 | 2.41 |
| Age range (months) | <0.033-0.03 | 0.07-0.93 | 1 - 3 | 4- 11.5 | <0.033-11.5 |
