# Supplementary material for: Early‐life regional and temporal variation in filaggrin‐derived natural moisturizing factor, filaggrin‐processing enzyme activity, corneocyte phenotypes and plasmin activity: implications for atopic dermatitis
Source: Br J Dermatol. 2018 Jun 29;179(2):431–41. doi: 10.1111/bjd.16691 (PMC6175251; doi:10.1111/bjd.16691)
Supplement: Supplementary file 7 — Fig S5. Bleomycin hydrolase (BH), calpain‐1 (C‐1) and plasmin activities in the stratum corneum (SC) of children (median with interquartile range) across two body regions in the following three age groups: < 48 h (n = 10); 48 h to 4 weeks (n = 10) and 1–11 months (n = 16). [file BJD-179-431-s007.docx]

|  | FLG WT | FLG HET | Unknown |
| --- | --- | --- | --- |
| Phase I | 118 | 11 | 0 |
|  |  |  |  |
| Phase II | 46 | 1 | 12 |
