# Supplementary material for: Early‐life regional and temporal variation in filaggrin‐derived natural moisturizing factor, filaggrin‐processing enzyme activity, corneocyte phenotypes and plasmin activity: implications for atopic dermatitis
Source: Br J Dermatol. 2018 Jun 29;179(2):431–41. doi: 10.1111/bjd.16691 (PMC6175251; doi:10.1111/bjd.16691)
Supplement: Supplementary file 8 — Powerpoint S1. Journal Club Slide Set. [file BJD-179-431-s008.pptx]

## Slide 1
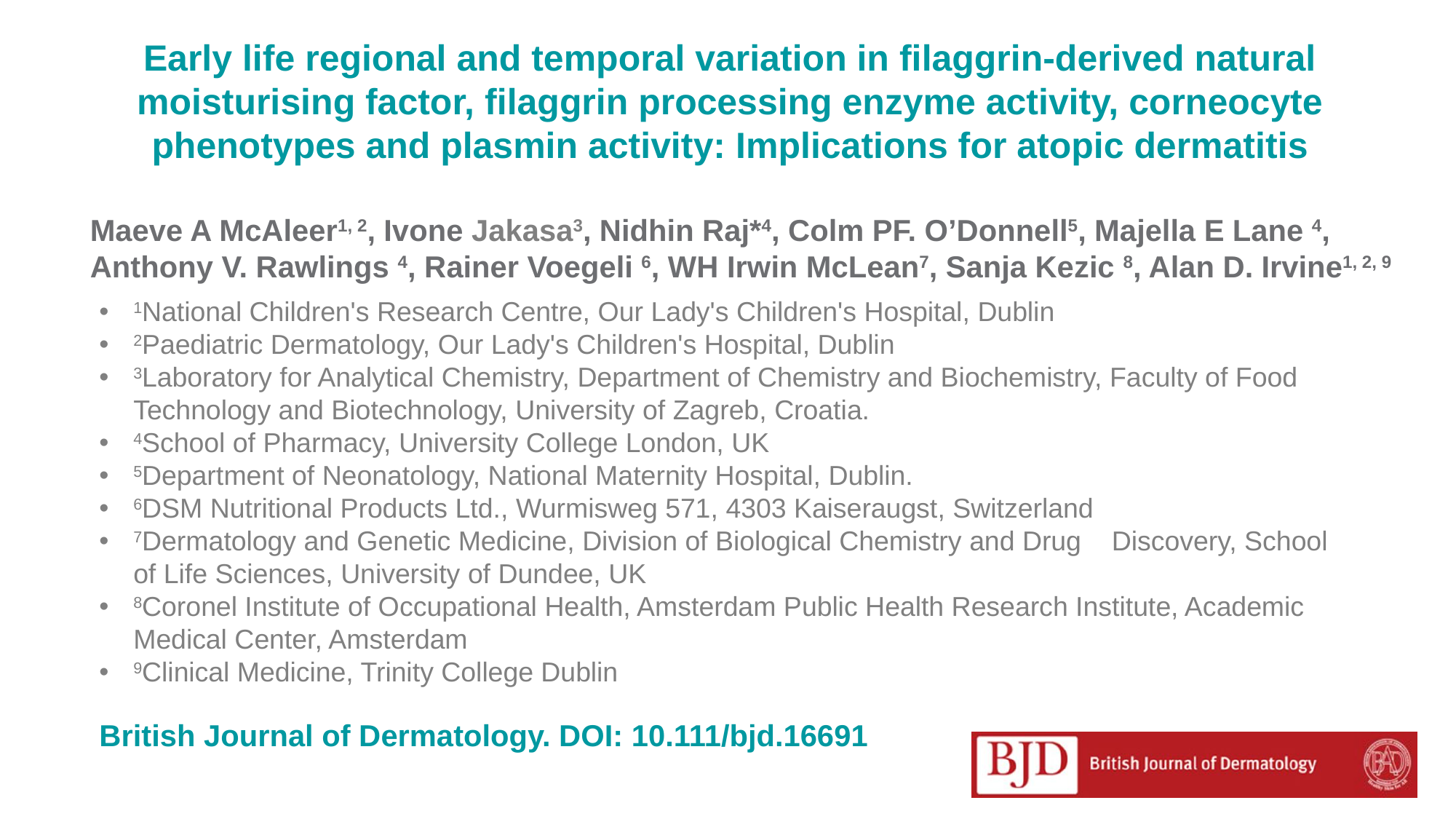

Early life regional and temporal variation in filaggrin-derived natural moisturising factor, filaggrin processing enzyme activity, corneocyte phenotypes and plasmin activity: Implications for atopic dermatitis
Maeve A McAleer1, 2, Ivone Jakasa3, Nidhin Raj*4, Colm PF. O’Donnell5, Majella E Lane 4, Anthony V. Rawlings 4, Rainer Voegeli 6, WH Irwin McLean7, Sanja Kezic 8, Alan D. Irvine1, 2, 9
1National Children's Research Centre, Our Lady's Children's Hospital, Dublin
2Paediatric Dermatology, Our Lady's Children's Hospital, Dublin
3Laboratory for Analytical Chemistry, Department of Chemistry and Biochemistry, Faculty of Food Technology and Biotechnology, University of Zagreb, Croatia.
4School of Pharmacy, University College London, UK
5Department of Neonatology, National Maternity Hospital, Dublin.
6DSM Nutritional Products Ltd., Wurmisweg 571, 4303 Kaiseraugst, Switzerland
7Dermatology and Genetic Medicine, Division of Biological Chemistry and Drug Discovery, School of Life Sciences, University of Dundee, UK
8Coronel Institute of Occupational Health, Amsterdam Public Health Research Institute, Academic Medical Center, Amsterdam
9Clinical Medicine, Trinity College Dublin
British Journal of Dermatology. DOI: 10.111/bjd.16691

## Slide 2
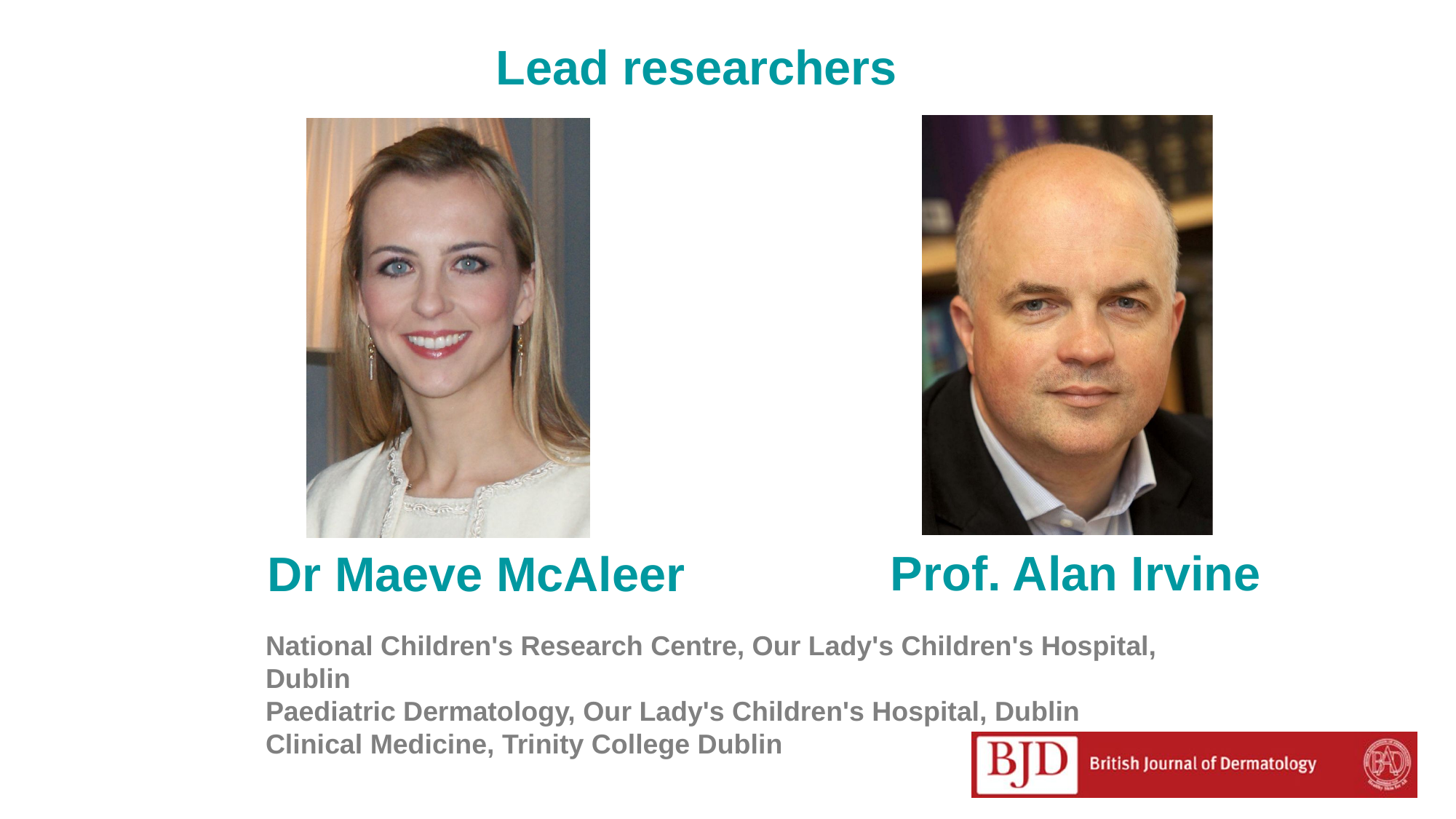

Lead researchers
Prof. Alan Irvine
# Dr Maeve McAleer
National Children's Research Centre, Our Lady's Children's Hospital, Dublin
Paediatric Dermatology, Our Lady's Children's Hospital, Dublin
Clinical Medicine, Trinity College Dublin

## Slide 3
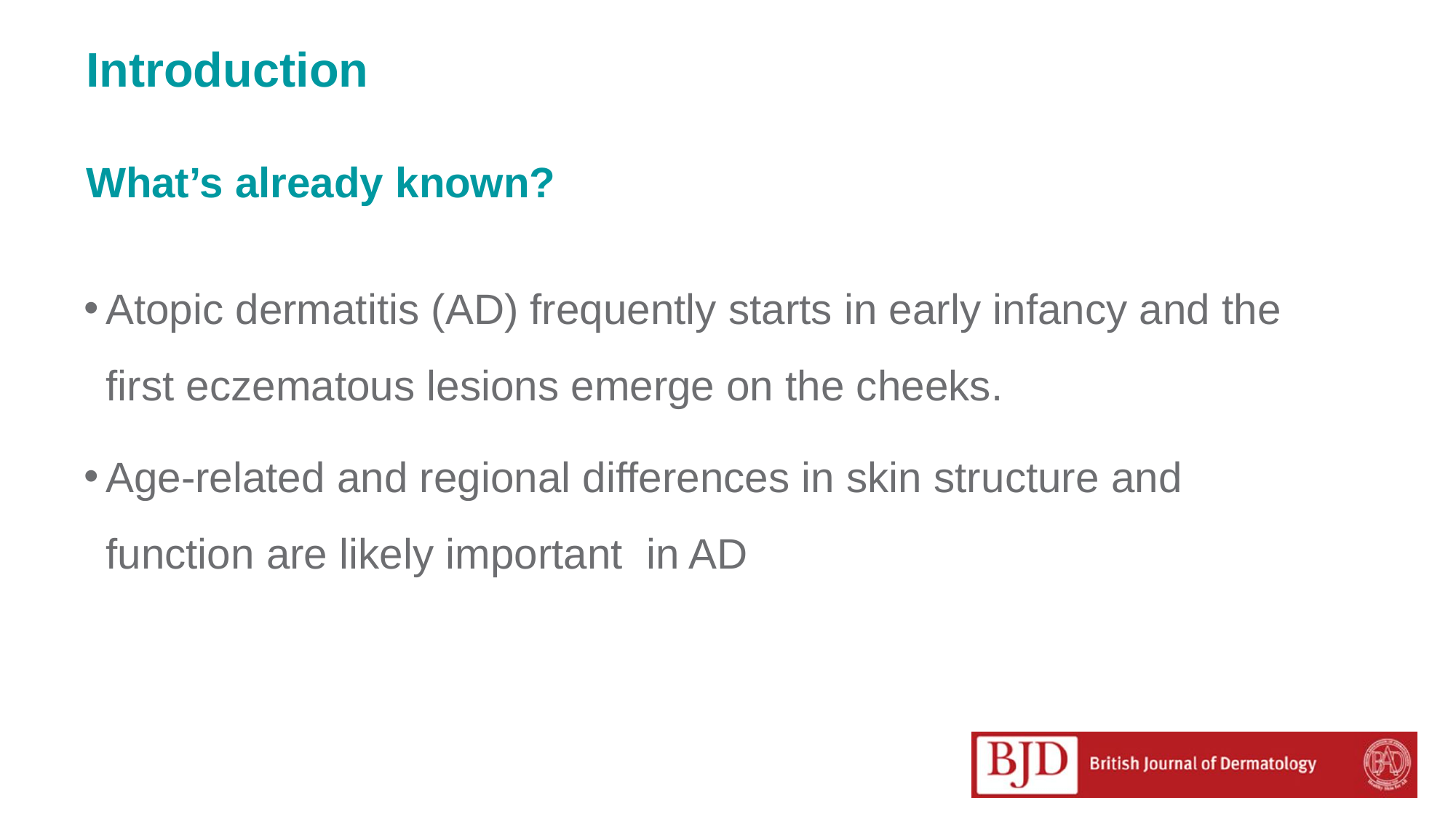

# Introduction What’s already known?
Atopic dermatitis (AD) frequently starts in early infancy and the first eczematous lesions emerge on the cheeks.
Age-related and regional differences in skin structure and function are likely important in AD

## Slide 4
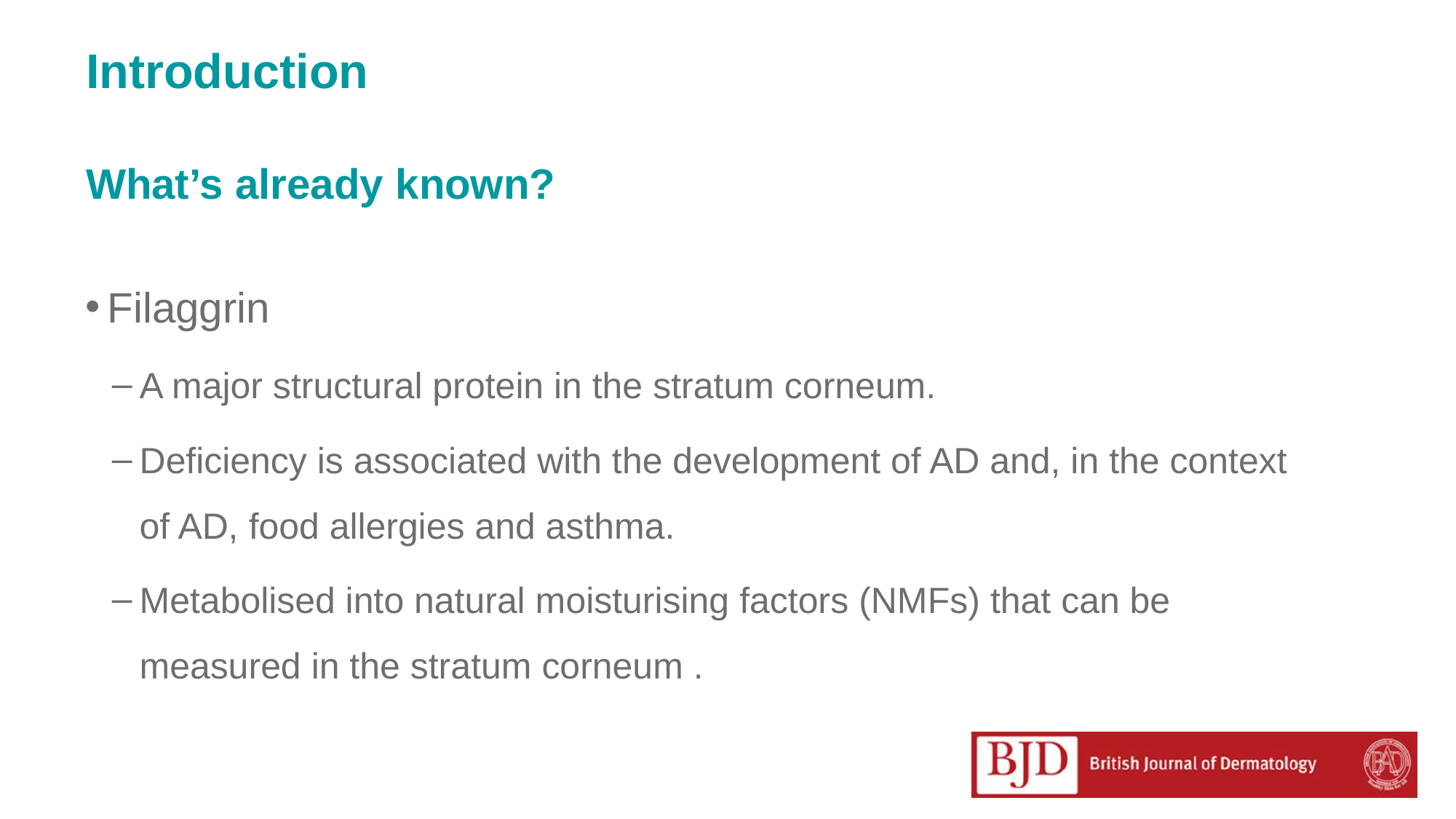

# Introduction What’s already known?
Filaggrin
A major structural protein in the stratum corneum.
Deficiency is associated with the development of AD and, in the context of AD, food allergies and asthma.
Metabolised into natural moisturising factors (NMFs) that can be measured in the stratum corneum .

## Slide 5
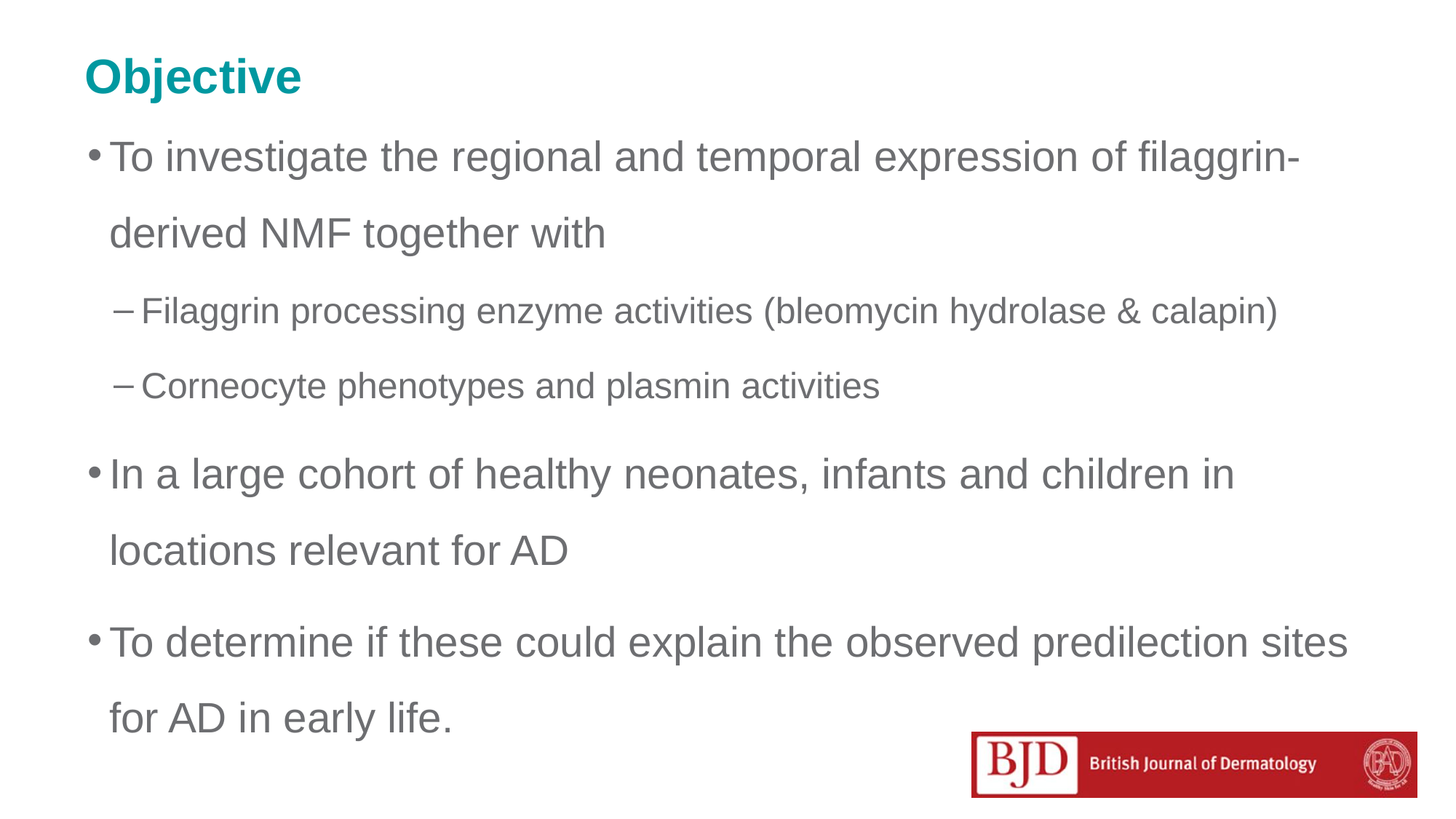

# Objective
To investigate the regional and temporal expression of filaggrin-derived NMF together with
Filaggrin processing enzyme activities (bleomycin hydrolase & calapin)
Corneocyte phenotypes and plasmin activities
In a large cohort of healthy neonates, infants and children in locations relevant for AD
To determine if these could explain the observed predilection sites for AD in early life.

## Slide 6
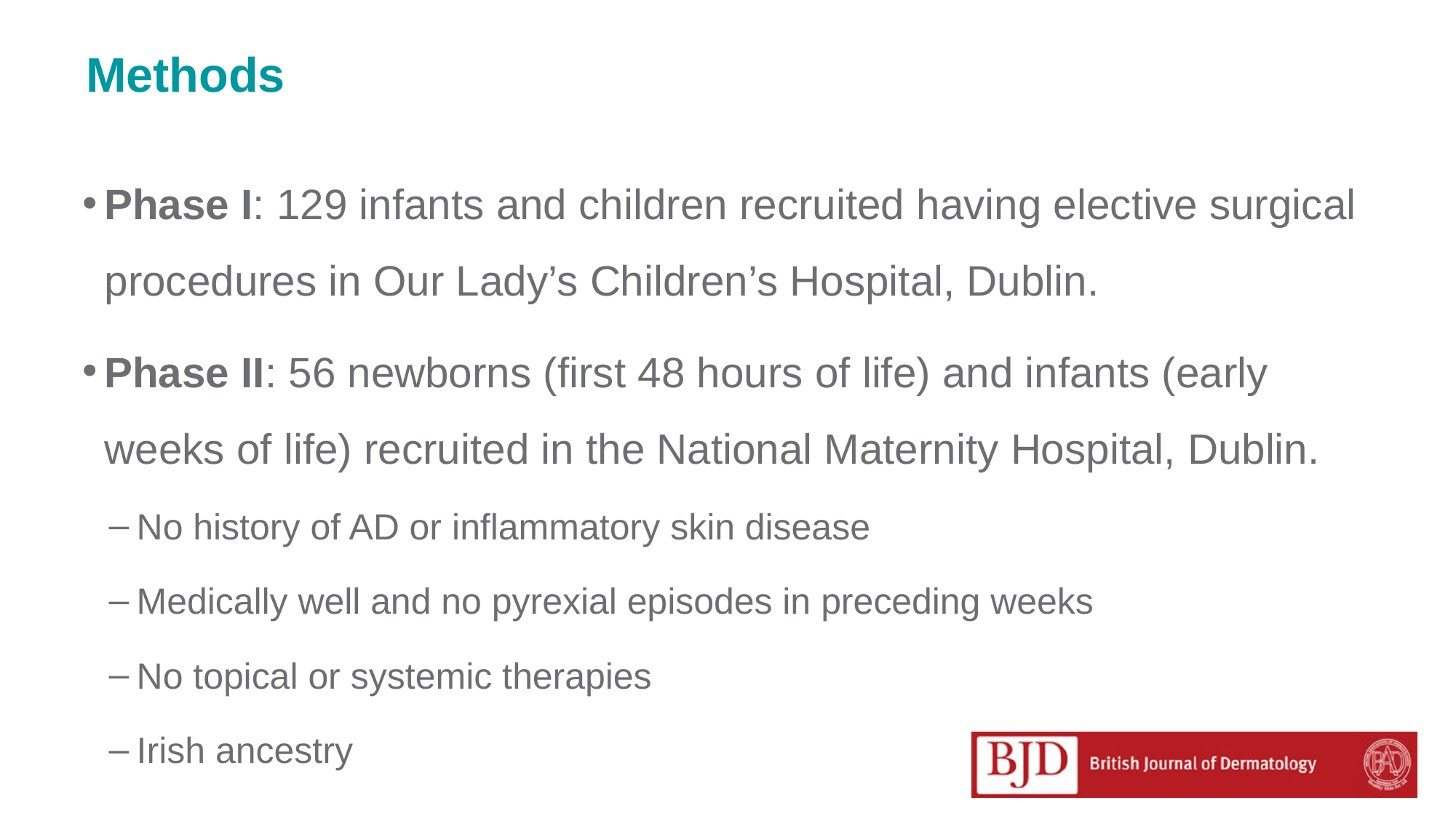

# Methods
Phase I: 129 infants and children recruited having elective surgical procedures in Our Lady’s Children’s Hospital, Dublin.
Phase II: 56 newborns (first 48 hours of life) and infants (early weeks of life) recruited in the National Maternity Hospital, Dublin.
No history of AD or inflammatory skin disease
Medically well and no pyrexial episodes in preceding weeks
No topical or systemic therapies
Irish ancestry

## Slide 7
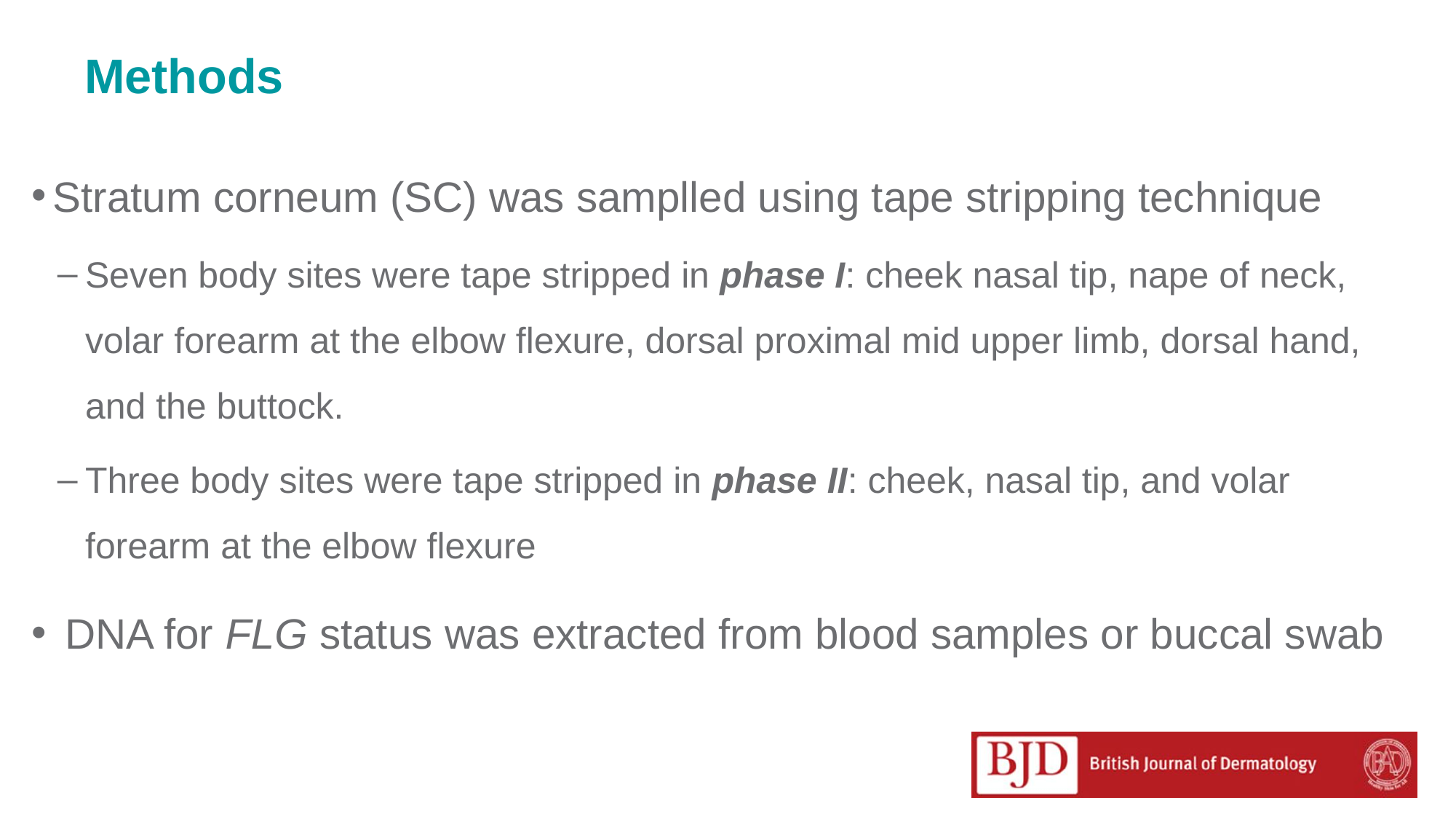

# Methods
Stratum corneum (SC) was samplled using tape stripping technique
Seven body sites were tape stripped in phase I: cheek nasal tip, nape of neck, volar forearm at the elbow flexure, dorsal proximal mid upper limb, dorsal hand, and the buttock.
Three body sites were tape stripped in phase II: cheek, nasal tip, and volar forearm at the elbow flexure
 DNA for FLG status was extracted from blood samples or buccal swab

## Slide 8
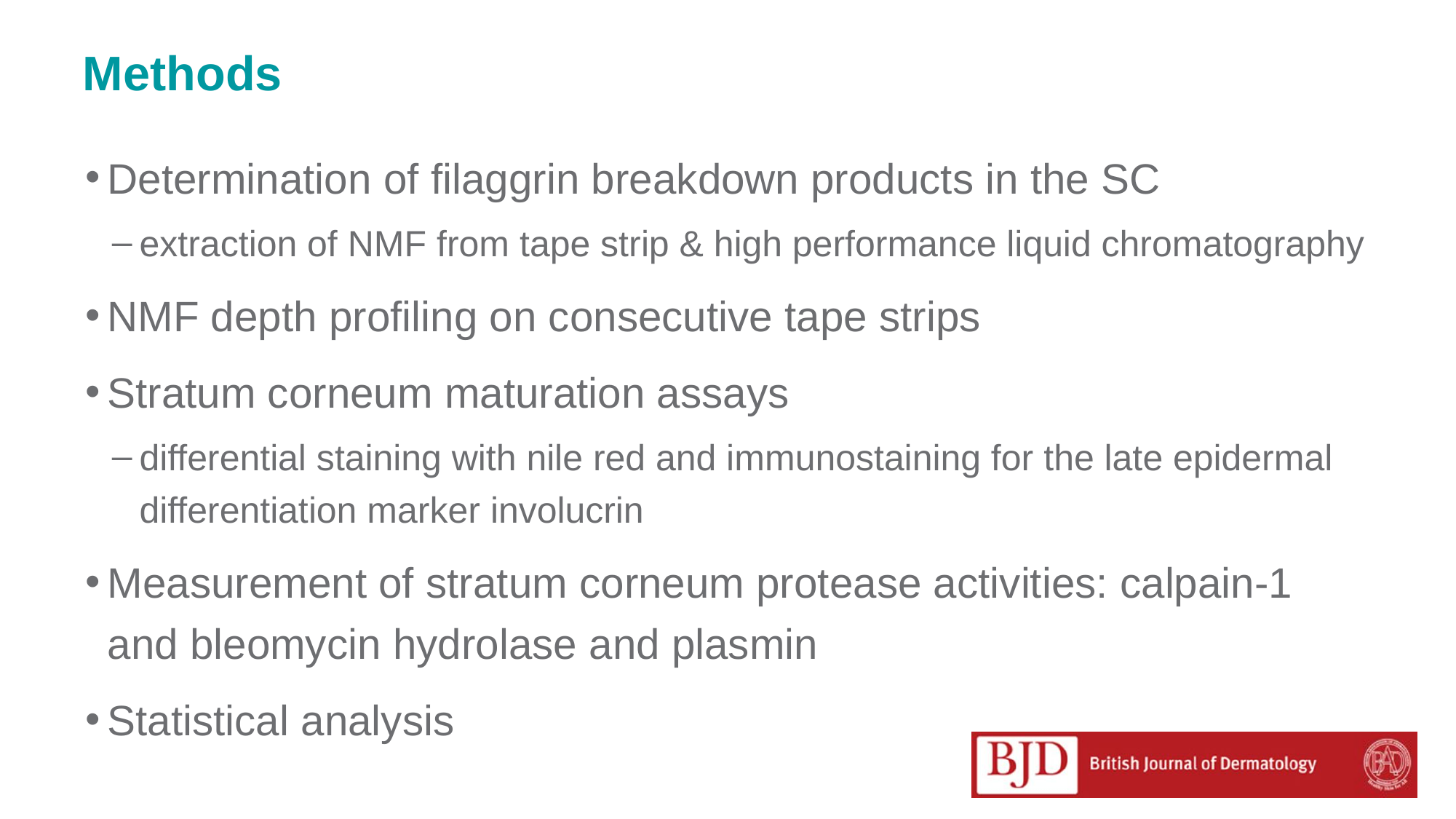

# Methods
Determination of filaggrin breakdown products in the SC
extraction of NMF from tape strip & high performance liquid chromatography
NMF depth profiling on consecutive tape strips
Stratum corneum maturation assays
differential staining with nile red and immunostaining for the late epidermal differentiation marker involucrin
Measurement of stratum corneum protease activities: calpain-1 and bleomycin hydrolase and plasmin
Statistical analysis

## Slide 9
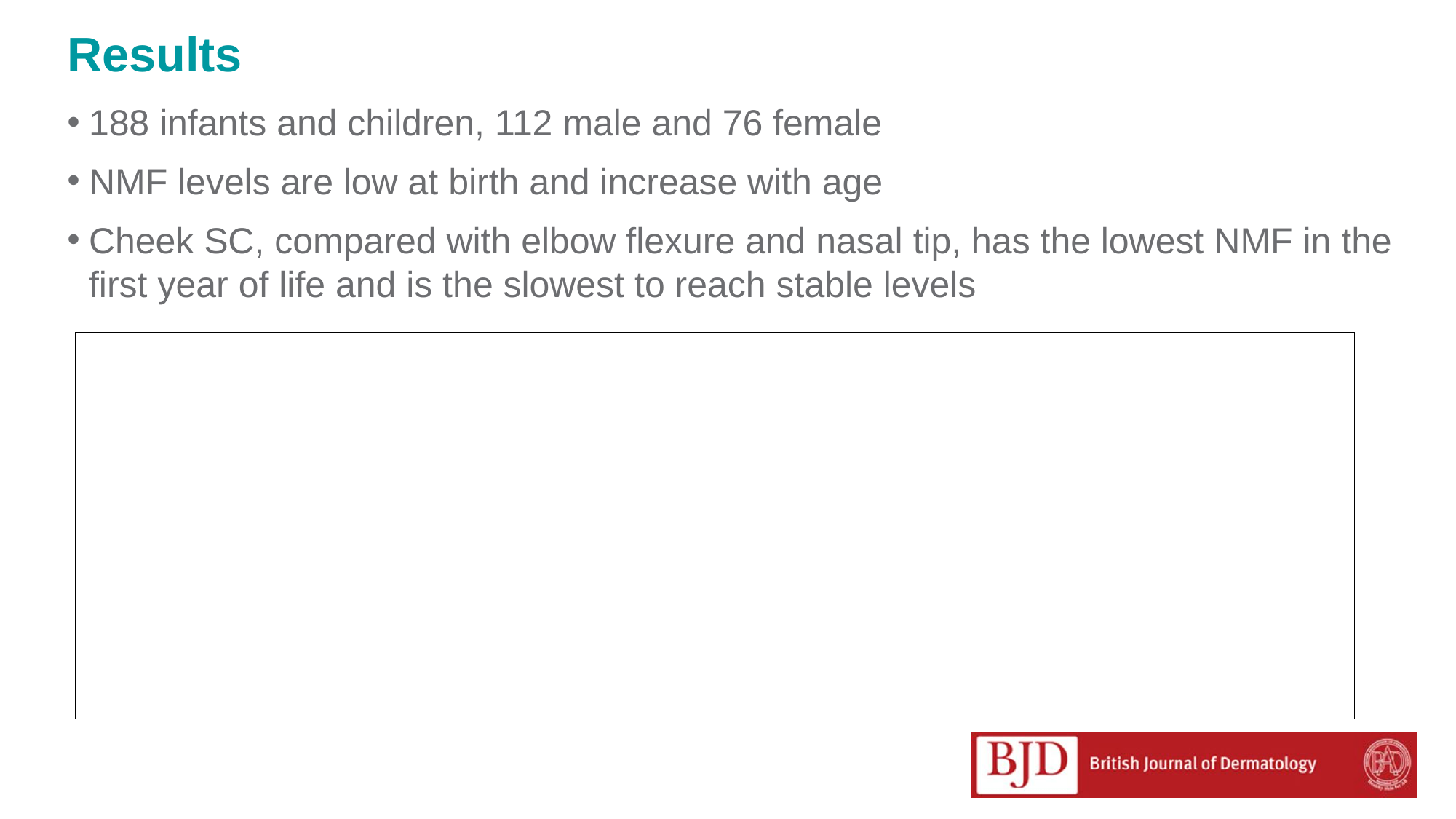

# Results
188 infants and children, 112 male and 76 female
NMF levels are low at birth and increase with age
Cheek SC, compared with elbow flexure and nasal tip, has the lowest NMF in the first year of life and is the slowest to reach stable levels

## Slide 10
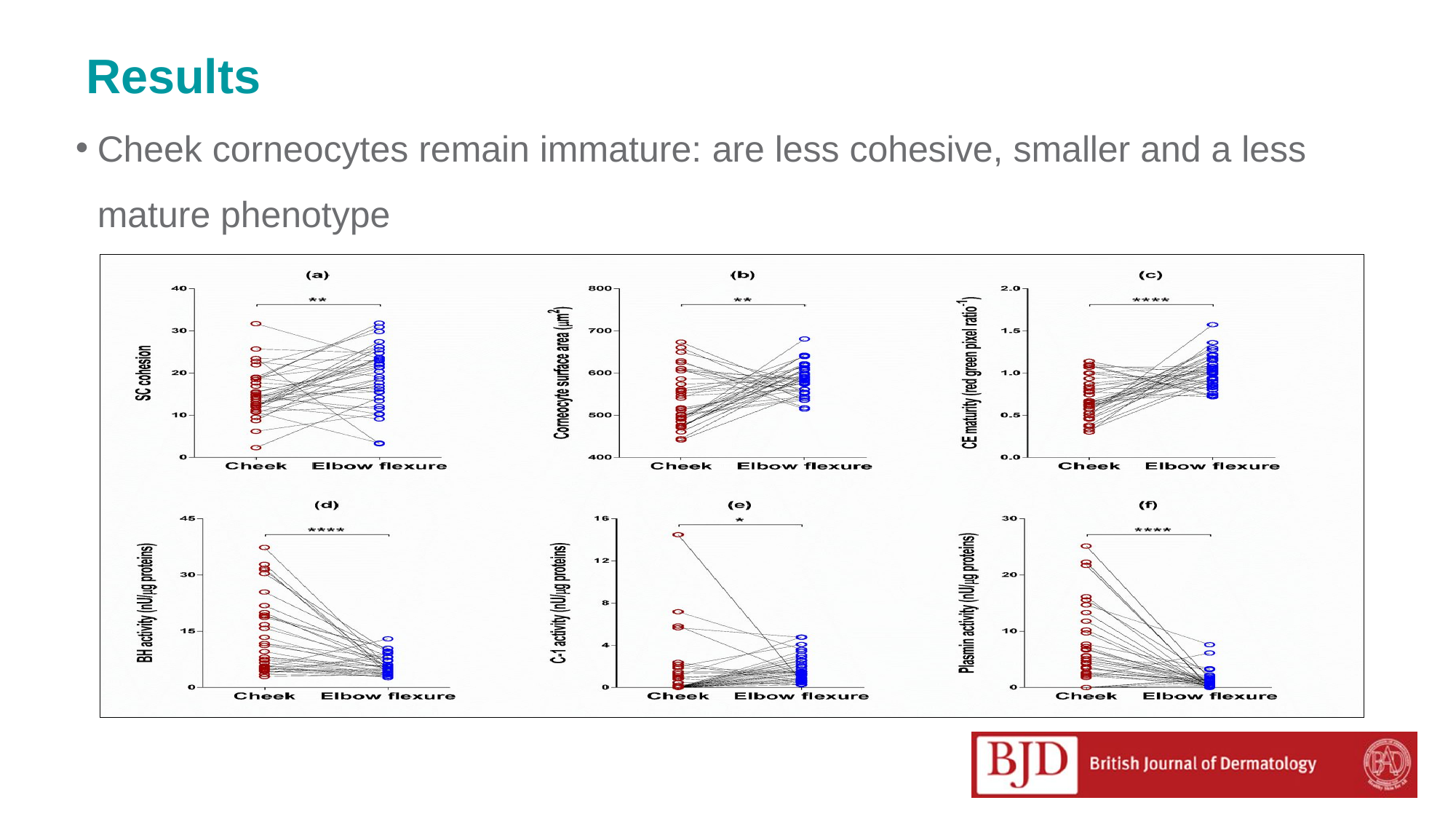

# Results
Cheek corneocytes remain immature: are less cohesive, smaller and a less mature phenotype

## Slide 11
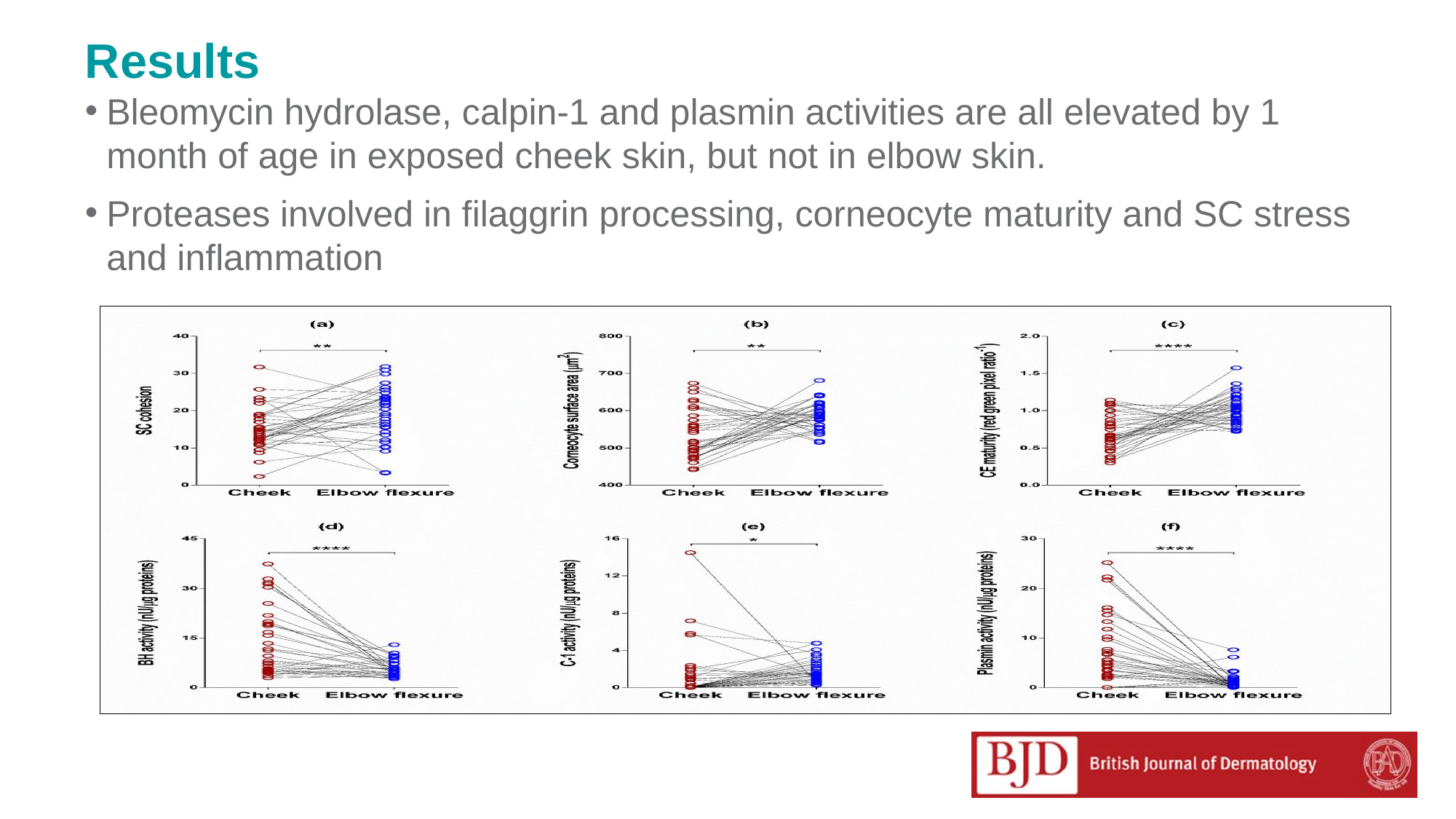

# Results
Bleomycin hydrolase, calpin-1 and plasmin activities are all elevated by 1 month of age in exposed cheek skin, but not in elbow skin.
Proteases involved in filaggrin processing, corneocyte maturity and SC stress and inflammation

## Slide 12
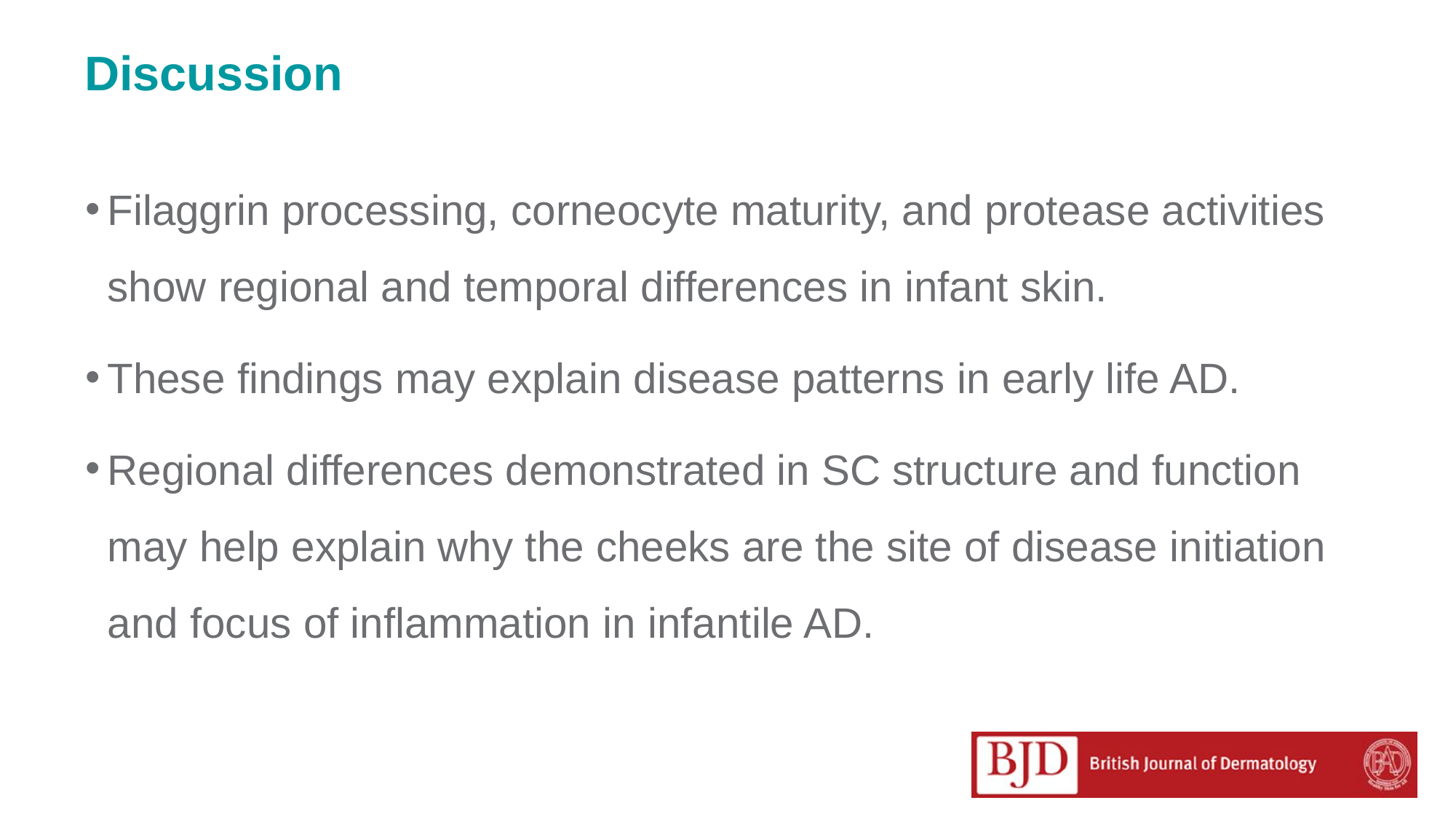

# Discussion
Filaggrin processing, corneocyte maturity, and protease activities show regional and temporal differences in infant skin.
These findings may explain disease patterns in early life AD.
Regional differences demonstrated in SC structure and function may help explain why the cheeks are the site of disease initiation and focus of inflammation in infantile AD.

## Slide 13
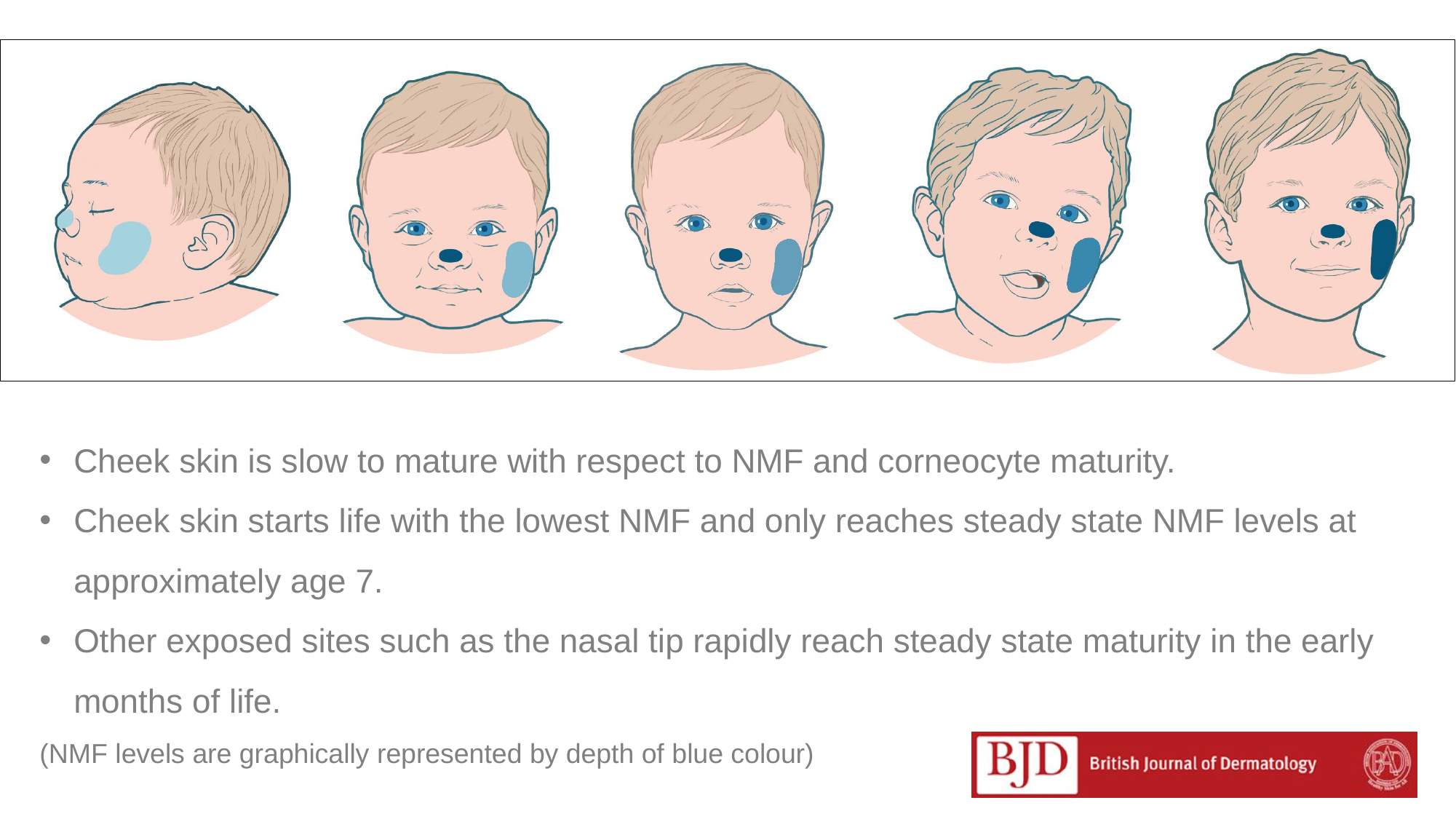

Cheek skin is slow to mature with respect to NMF and corneocyte maturity.
Cheek skin starts life with the lowest NMF and only reaches steady state NMF levels at approximately age 7.
Other exposed sites such as the nasal tip rapidly reach steady state maturity in the early months of life.
(NMF levels are graphically represented by depth of blue colour)

## Slide 14
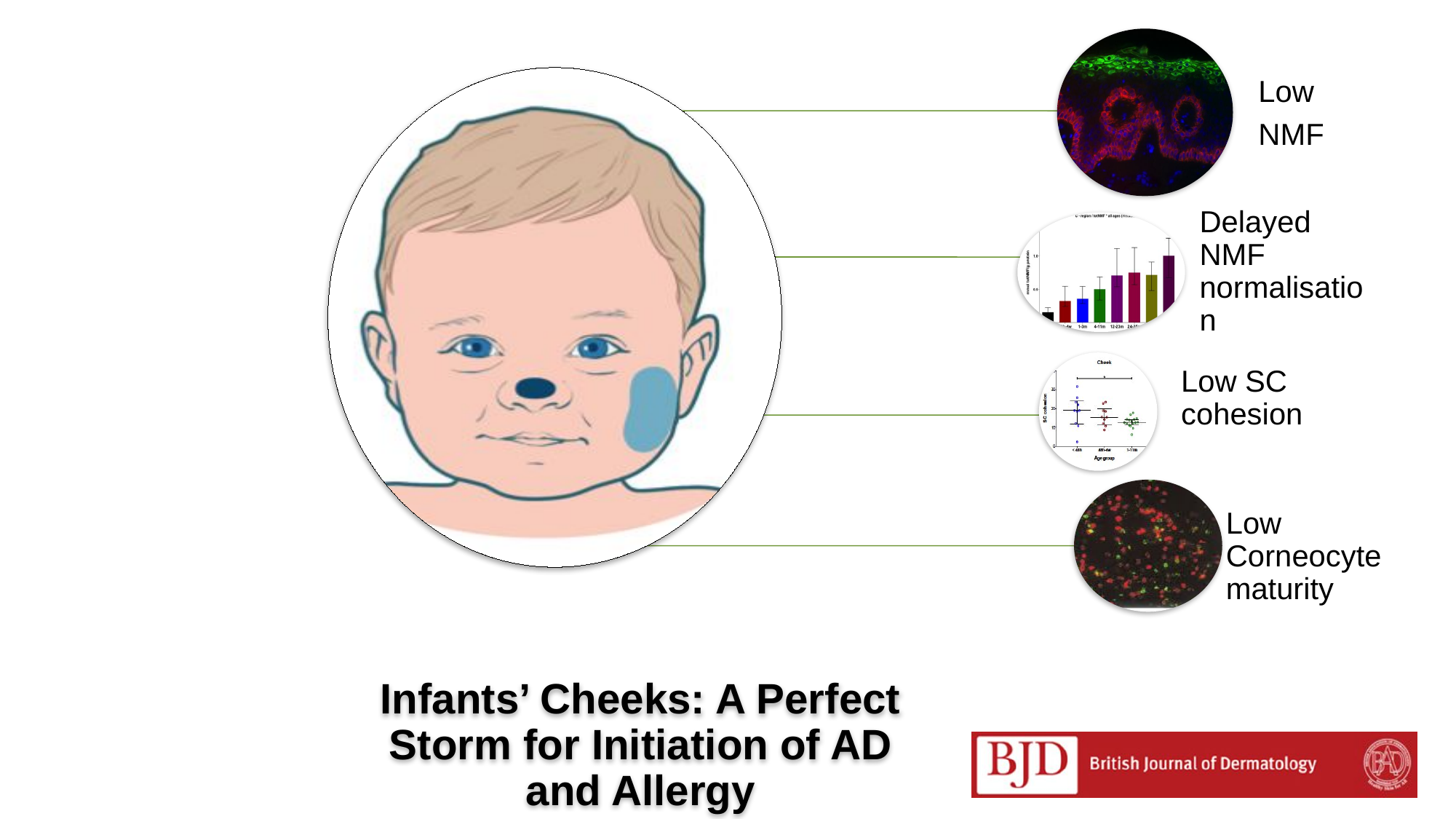

## Slide 15
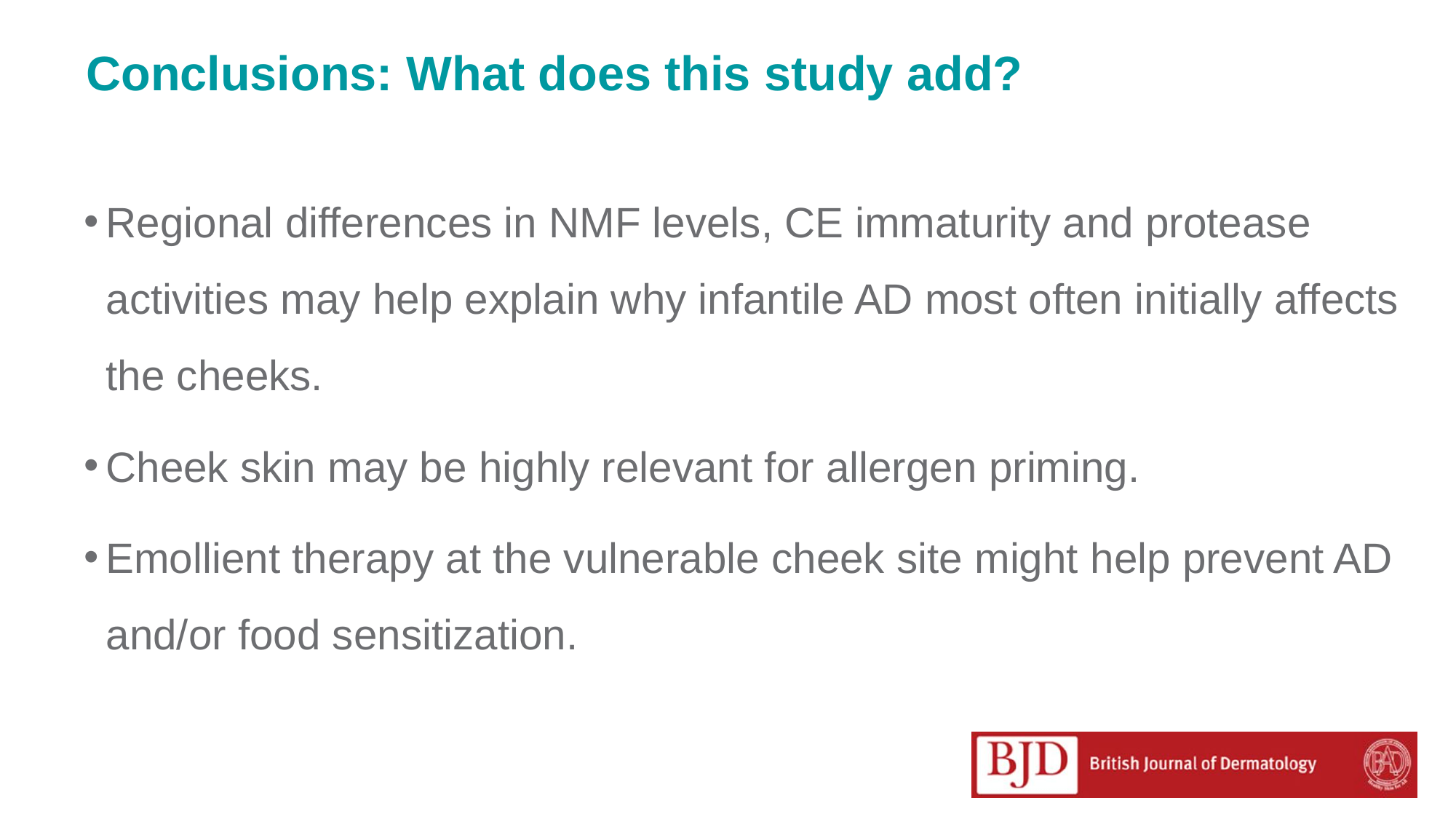

# Conclusions: What does this study add?
Regional differences in NMF levels, CE immaturity and protease activities may help explain why infantile AD most often initially affects the cheeks.
Cheek skin may be highly relevant for allergen priming.
Emollient therapy at the vulnerable cheek site might help prevent AD and/or food sensitization.

## Slide 16
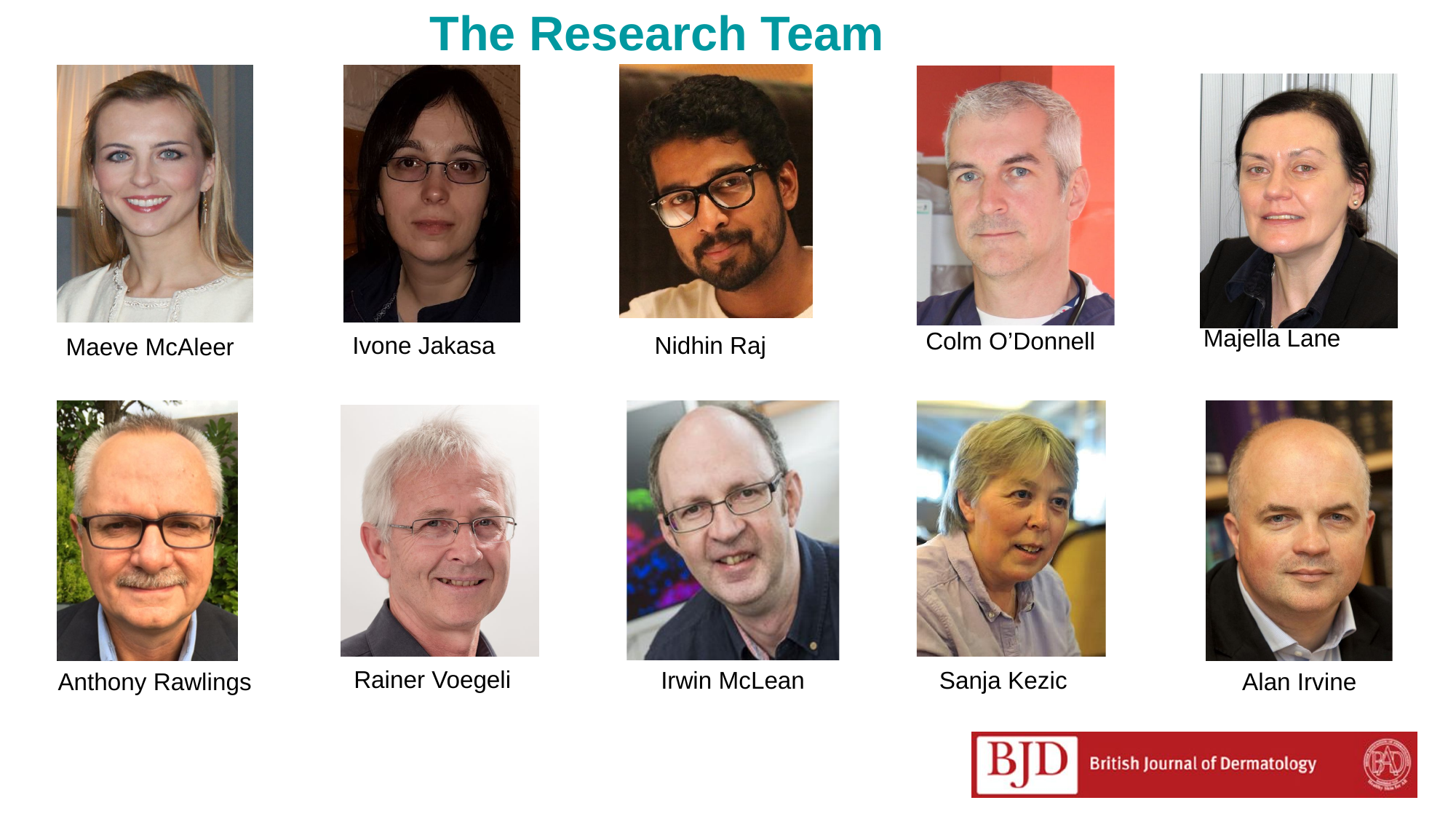

# The Research Team
Majella Lane
Colm O’Donnell
Nidhin Raj
Ivone Jakasa
Maeve McAleer
Rainer Voegeli
Irwin McLean
Sanja Kezic
Anthony Rawlings
Alan Irvine

## Slide 17
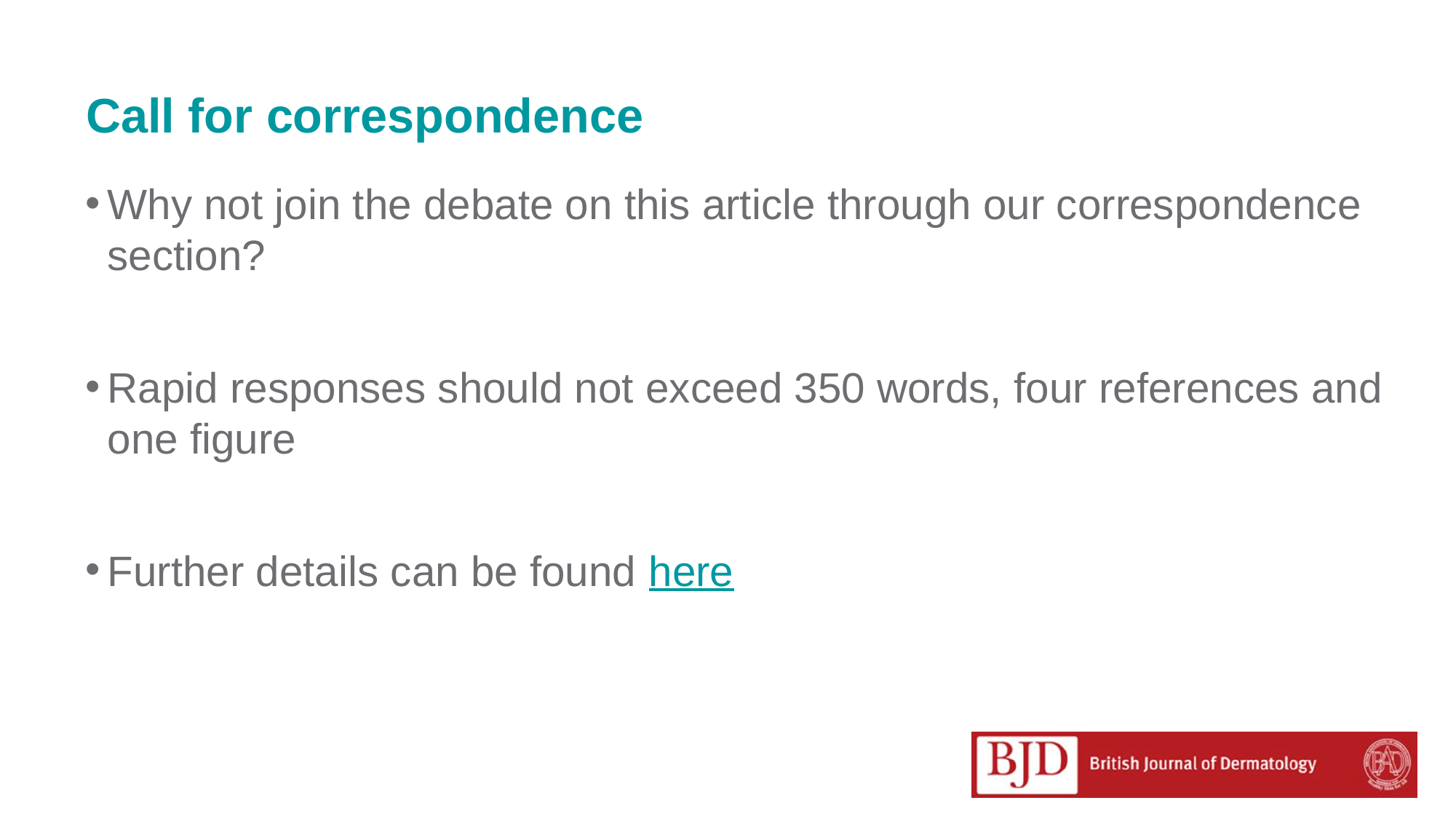

# Call for correspondence
Why not join the debate on this article through our correspondence section?
Rapid responses should not exceed 350 words, four references and one figure
Further details can be found here
