# Supplementary material for: Early‐life regional and temporal variation in filaggrin‐derived natural moisturizing factor, filaggrin‐processing enzyme activity, corneocyte phenotypes and plasmin activity: implications for atopic dermatitis
Source: Br J Dermatol. 2018 Jun 29;179(2):431–41. doi: 10.1111/bjd.16691 (PMC6175251; doi:10.1111/bjd.16691)
Supplement: Supplementary file 5 — Fig S3. Levels of total natural moisturizing factor (NMF), histidine, pyrrolidone carboxylic acid (PCA) and sum of trans‐ and cis‐urocanic acid (UCA) in the stratum corneum (SC) of children (median with interquartile range) across four body regions in the following different age groups: 1–11 months (n = 25); 12–35 months (n = 25) and 36–72 months (n = 27). [file BJD-179-431-s005.pdf]

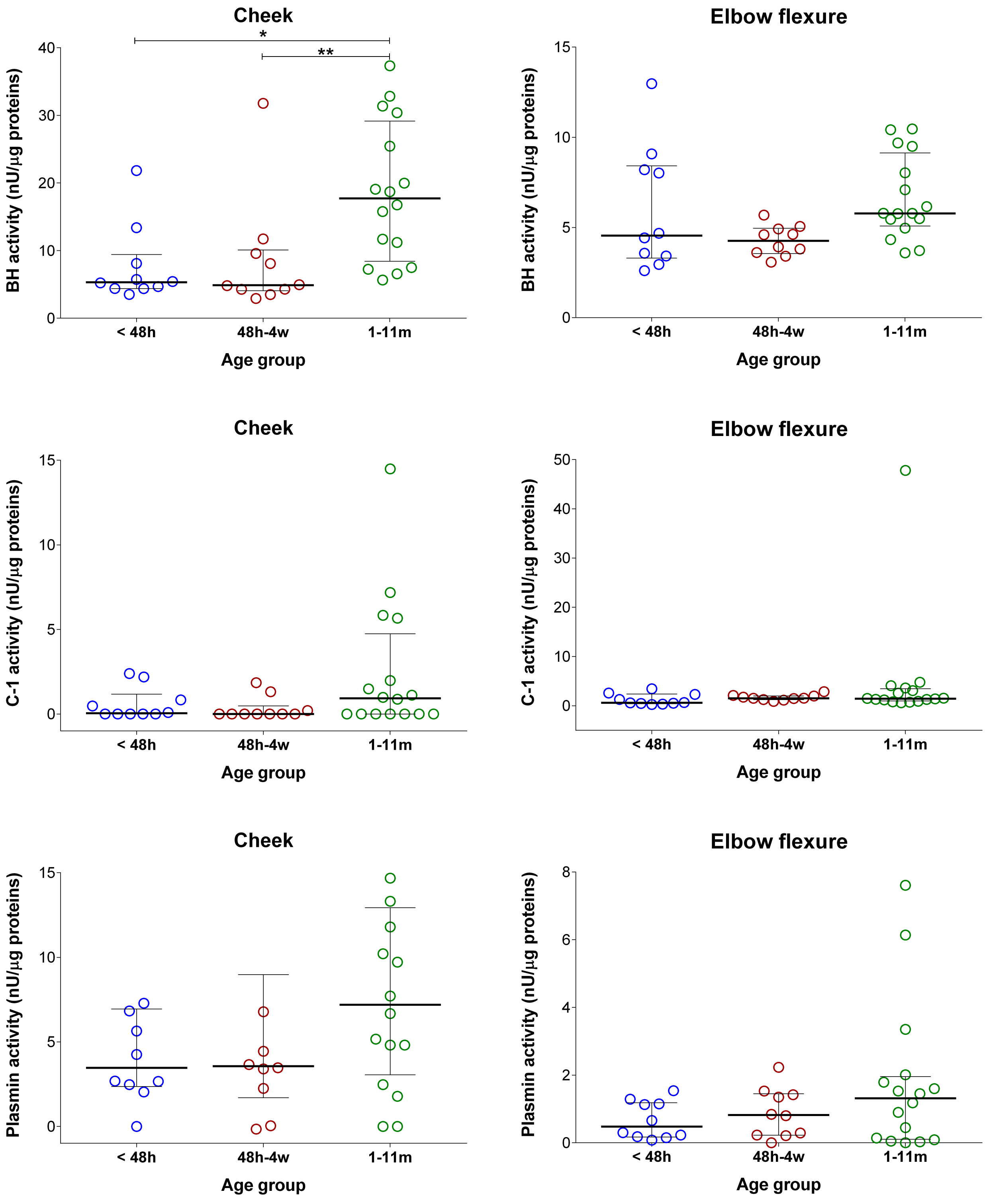
Cheek
*
**
BH activity (nU/µg proteins)
0
10
20
30
40
< 48h
48h-4w
1-11m
Age group
Elbow flexure
BH activity (nU/µg proteins)
0
5
10
15
< 48h
48h-4w
1-11m
Age group
Cheek
C-1 activity (nU/µg proteins)
0
5
10
15
< 48h
48h-4w
1-11m
Age group
Elbow flexure
C-1 activity (nU/µg proteins)
0
10
20
30
40
50
< 48h
48h-4w
1-11m
Age group
Cheek
Plasmin activity (nU/µg proteins)
0
5
10
15
< 48h
48h-4w
1-11m
Age group
Elbow flexure
Plasmin activity (nU/µg proteins)
0
2
4
6
8
< 48h
48h-4w
1-11m
Age group
